# An Abundant and Diverse New Family of Electron Bifurcating Enzymes With a Non-canonical Catalytic Mechanism

**DOI:** 10.3389/fmicb.2022.946711

**Published:** 2022-07-08

**Authors:** Gerrit J. Schut, Dominik K. Haja, Xiang Feng, Farris L. Poole, Huilin Li, Michael W. W. Adams

**Affiliations:** ^1^Department of Biochemistry and Molecular Biology, University of Georgia, Athens, GA, United States; ^2^Department of Structural Biology, Van Andel Institute, Grand Rapids, MI, United States

**Keywords:** electron bifurcation/confurcation, bioenergenesis, hydrogenase, ferredoxin, flavin, iron–sulfur cluster, HydABC

## Abstract

Microorganisms utilize electron bifurcating enzymes in metabolic pathways to carry out thermodynamically unfavorable reactions. Bifurcating FeFe-hydrogenases (HydABC) reversibly oxidize NADH (E′∼−280 mV, under physiological conditions) and reduce protons to H_2_ gas (E°′−414 mV) by coupling this endergonic reaction to the exergonic reduction of protons by reduced ferredoxin (Fd) (E′∼−500 mV). We show here that HydABC homologs are surprisingly ubiquitous in the microbial world and are represented by 57 phylogenetically distinct clades but only about half are FeFe-hydrogenases. The others have replaced the hydrogenase domain with another oxidoreductase domain or they contain additional subunits, both of which enable various third reactions to be reversibly coupled to NAD^+^ and Fd reduction. We hypothesize that all of these enzymes carry out electron bifurcation and that their third substrates can include hydrogen peroxide, pyruvate, carbon monoxide, aldehydes, aryl-CoA thioesters, NADP^+^, cofactor F_420_, formate, and quinones, as well as many yet to be discovered. Some of the enzymes are proposed to be integral membrane-bound proton-translocating complexes. These different functionalities are associated with phylogenetically distinct clades and in many cases with specific microbial phyla. We propose that this new and abundant class of electron bifurcating enzyme be referred to as the Bfu family whose defining feature is a conserved bifurcating BfuBC core. This core contains FMN and six iron sulfur clusters and it interacts directly with ferredoxin (Fd) and NAD(H). Electrons to or from the third substrate are fed into the BfuBC core *via* BfuA. The other three known families of electron bifurcating enzyme (abbreviated as Nfn, EtfAB, and HdrA) contain a special FAD that bifurcates electrons to high and low potential pathways. The Bfu family are proposed to use a different electron bifurcation mechanism that involves a combination of FMN and three adjacent iron sulfur clusters, including a novel [2Fe-2S] cluster with pentacoordinate and partial non-Cys coordination. The absolute conservation of the redox cofactors of BfuBC in all members of the Bfu enzyme family indicate they have the same non-canonical mechanism to bifurcate electrons. A hypothetical catalytic mechanism is proposed as a basis for future spectroscopic analyses of Bfu family members.

## Introduction

Electron bifurcation is an energy coupling process in biology that enables thermodynamically unfavorable reactions to occur that are not dependent on ATP hydrolysis or driven by an ion gradient (Buckel and Thauer, 2018a,b; Buckel, 2021). In this process, a single enzyme reversibly couples an endergonic reaction (X → A, non-spontaneous) to an exergonic reaction (X → B, spontaneous) to generate a net reversible reaction (2X ↔ A + B) with minimal free energy change and maximal energy conservation. In essence, an exergonic reaction is used to drive an endergonic one. Electron bifurcating enzymes therefore interact with three different substrates, generating two products (B, C) from a single substrate (2A) or, in the reverse confurcating reaction, one product (2A) from two substrates (B, C). Electron bifurcation is now recognized as a major energy converting system in biology (Buckel and Thauer, 2013; Peters et al., 2016). Electron bifurcating enzymes are ubiquitous in anaerobic bacteria and are even utilized by some aerobes (Poudel et al., 2018).

Electron bifurcation is providing a unifying explanation for many enigmatic reactions in microbial metabolism (Buckel and Thauer, 2018b; Buckel, 2021), such as how NADH [E′ = −280 mV under physiological conditions (Buckel and Thauer, 2018a)] is used to generate the reduced form of the very low potential iron sulfur redox protein, Fd [E′∼−500 mV (Buckel and Thauer, 2018a)], which is used to drive many key microbial processes that NADH is not able to do, such as H_2_ and methane production and N_2_ fixation. This was solved by Buckel and Thauer who showed that in some Clostridia, the cytoplasmic enzyme Etf/butyryl-CoA dehydrogenase catalyzed the reduction of Fd by NADH by simultaneously coupling it to the reduction of crotonyl-CoA (E_m_ −10 mV) by NADH (Li et al., 2008). Several enzymes of the electron bifurcating EtfAB family are now known. These include the Etf/FixABCX complex, which enables NADH to reduce Fd by coupling the reaction to the exergonic reduction of menaquinone (E_m_ −74 mV) by NADH (Bertsch and Muller, 2015; Weghoff et al., 2015; Ledbetter et al., 2017; Schut et al., 2019; Feng et al., 2021).

Two other phylogenetically unrelated groups of flavin-based electron bifurcating (FBEB) enzymes are known, NfnAB and Hdr-Mvh complexes. NfnAB enables NADH and Fd to be used simultaneously for the reduction of NADP^+^ [E_m_ ∼−380 mV, physiological conditions (Buckel and Thauer, 2018a)] to drive biosynthesis (Demmer et al., 2015; Lubner et al., 2017), while the Hdr-Mvh complex, which contains hydrogenase (MvhAGD) and heterodisulfide reductase (HdrABC), uses H_2_ (E°′ = −414 mV) to reduce a heterodisulfide (E_m_ = −140 mV) and Fd (Kaster et al., 2011). In addition, it was recently shown that HdrA modules are highly abundant and do not occur only in a complex with MvH (Appel et al., 2021). All three of these electron bifurcating enzyme complexes containing EtfAB, NfnAB, or HdrA, contain an unusual flavin, designated BF-FAD, that is the site of electron bifurcation. This flavin is coordinated such that its half potentials are highly crossed and low potential electrons are guided through a low potential pathway ultimately reducing Fd while the high potential electrons are directed to a high potential pathway reducing the high potential acceptor (Chowdhury et al., 2014; Demmer et al., 2015, 2018; Lubner et al., 2017; Schut et al., 2019).

The fourth type of electron bifurcating enzyme is represented by trimeric FeFe-hydrogenases that simultaneously couple endergonic NADH oxidation to the exergonic oxidation of reduced Fd to generate H_2_ gas (Eq. 1, where Fd is a two-electron carrier). This enables microorganisms to convert glucose to H_2_, CO_2_ and acetate without generating other more reduced


(1) $${\text{Fd}}_{\text{red}}+\text{NADH}+3\,H^{+}↔2\,H_{2}+{\text{Fd}}_{\text{ox}}+\text{NAD}$$

carbon compounds, such as ethanol, lactate, or butyrate. The trimeric bifurcating FeFe-hydrogenase (FeFe-HydABC) was discovered in the extremely thermophilic bacterium (T_max_ 90°C), *Thermotoga maritima* (Schut and Adams, 2009), and has since been purified from several mesophilic species (Schuchmann and Muller, 2012; Wang et al., 2013c; Zheng et al., 2014). Their electron bifurcating mechanism, however, is not understood (Zuchan et al., 2021). While a special BF-FAD in the other three types of electron bifurcating enzyme accepts electrons from the mid potential donor and bifurcates them to high and low potential acceptors (Buckel and Thauer, 2018b), this is not the case in FeFe-hydrogenases based on current understanding of flavin chemistry. In its electron bifurcation reaction (reverse of physiological reaction in Eq. 1), the single FMN found in the hydrogenase must reduce the high potential acceptor (NAD^+^) by two electrons originating from the mid-potential donor (H_2_) generating a stable flavosemiquinone intermediate, and this is a thermodynamically favorable reaction. The flavin must also transfer single electrons to reduce the low potential acceptor, Fd, which, according to what is known about FBEB enzymes, involves a highly reactive and unstable flavosemiquinone. It is not clear how a single flavin can carry out both types of electron transfer reaction in the hydrogenase. Hence FMN plays a very complex role in FeFe-hydrogenases and this must be very different from the straightforward role of the BF-FADs in other three types of electron bifurcating enzyme.

**FIGURE 1:**
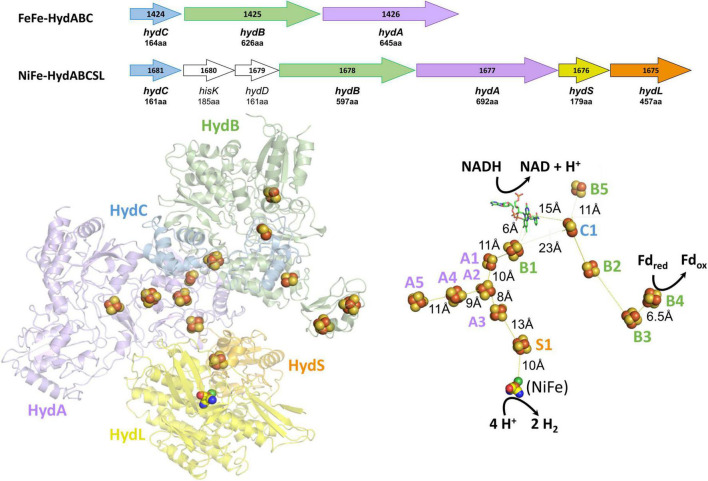
Operons, structures, and cofactor content of the *A. mobile* bifurcating NiFe-hydrogenase. *Upper*: the operons encoding *Thermotoga maritima* FeFe-HydABC and *A. mobile* NiFe-HydABCSL are shown where colors indicate homologous subunits. Numbers inside the arrow refer to the gene number and the numbers of amino acids encoded are indicated. The proteins encoded by *hydS* and *hydL* are homologs of non-bifurcating NiFe-hydrogenases but *hisK* and *hydD* are not part of the NiFe-holoenzyme. *Lower*: the cryoEM protein structure of pentameric NiFe-hydrogenase from *A. mobile* showing the cofactor content of the ABCSL subunits (adapted from Feng et al., 2022). The iron sulfur clusters are numbered according to the subunit that contains them. The distances between the C1, B2, and B3 clusters are not shown since, in contrast to the other distances shown, they vary during the electron bifurcation reaction. As discussed in the text, elsewhere in the manuscript the Hyd designation is replaced by Bfu.

Insights into the electron bifurcation mechanism of the FeFe-hydrogenases were recently provided by the cryo-EM structure of a related electron bifurcating NiFe-hydrogenase termed NiFe-HydABCSL (Feng et al., 2022). This enzyme was purified in its active electron bifurcating form from native biomass of the moderately thermophilic bacterium (T_max_ 60°C) *Acetomicrobium mobile*. In addition to homologs of the FeFe-HydABC subunits, where HydA lacks the catalytic H cluster of the FeFe-hydrogenases, the NiFe-enzyme also contains the small (S) and large (L) subunits of a conventional group 3 NiFe-hydrogenase (Figure 1). Like the FeFe-HydABC enzyme, NiFe-HydABCSL catalyzes a tightly coupled electron bifurcating reaction and reversibly oxidizes NADH and reduced Fd to generate H_2_ gas (Eq. 1). The structure of the NiFe-enzyme demonstrated that previous proposals for the site of electron bifurcation in FeFe hydrogenases are all very unlikely, as recently predicted from a bioinformatic analysis (Zuchan et al., 2021). Instead, based on the structure, the bifurcation of four electrons (Eq. 1) was proposed to be achieved by the combination of the single FMN in a unique arrangement of nearby iron sulfur clusters. Although the exact mechanism was not known, it was clear that significant conformational changes were involved and bifurcation ready and post bifurcation conformational states were identified (Feng et al., 2022).

We show here that homologs of the HydABC subunits found in the electron bifurcating *T. maritima* FeFe- and the *A. mobile* NiFe-hydrogenases are part of a new and remarkably abundant and diverse family of electron bifurcating enzyme that we designate as the BfuABC family. Surprisingly, only about half of them are hydrogenases. We hypothesize that all of the Bfu enzymes carry out electron bifurcation reactions using a highly conserved electron bifurcating “BfuBC” core architecture and that they all use NAD(H) and Fd as two of their substrates to catalyze a broad range of reactions involving a third substrate that is not H_2_. We predict that these third substrates can include hydrogen peroxide, pyruvate, carbon monoxide, aldehydes, aryl-CoA thioester derivatives, NADP^+^, cofactor F_420_, formate, and quinones, as well as many yet to be discovered. In addition, some of these Bfu enzymes are predicted to be integral membrane complexes capable of proton-pumping. Based on the structure of the bifurcating HydABCSL NiFe-hydrogenase (Feng et al., 2022), we have categorized the Bfu family members into four structural types based on their pathways of electron transfer to the bifurcating BfuBC core. Moreover, we hypothesize that they all use a new type of mechanism for electron bifurcation involving a single flavin in combination with three iron–sulfur clusters that is independent of the third substrate that they utilize. Members of this BfuABC enzyme family are involved in a large number of previously unknown energy-conserving metabolic functions in both Bacteria and Archaea.

## Phylogeny of the HydABC Family

Bifurcating FeFe-hydrogenases contain three subunits designated HydABC, where HydA contains the catalytic H cluster, the site of reversible H_2_ activation. Bifurcating NiFe-hydrogenases also contain homologs of HydABC, where the catalytic H cluster of the FeFe-enzyme is replaced by a [4Fe-4S] cluster, and one of the two additional subunits (HydSL) contain the catalytic NiFe-site (Feng et al., 2022). As shown in Figure 1, in the structurally characterized NiFe-enzyme, HydA and HydB each contain five iron–sulfur clusters, designated A1–A5 and B1–B5, respectively, while HydC contains a single cluster (C1). HydB also contains the flavin (FMN) that binds NAD(H) and this subunit also binds Fd (Feng et al., 2022). Herein we have carried out a detailed comparative genome analysis and found that the two HydABC subunits of these two hydrogenases are highly conserved in anaerobic microorganisms. Virtually all of the 1558 HydB sequences retrieved from the InterPro database (Blum et al., 2021) are encoded in operons that also encode HydC and HydA homologs, although in terms of domain structure the HydAs are much less conserved than HydB and HydC, as discussed below. Surprisingly, only about half of the HydABC genes are found in clusters predicted to encode FeFe- or NiFe-hydrogenases. In most cases, additional genes are present in the HydABC clusters that encode proteins that are predicted to catalyze reactions other than H_2_ production or oxidation. The HydABC family is therefore much more diverse than previously thought and is not limited to hydrogenases. Consequently, the Hyd designation is not appropriate. Hence we will refer to this proposed new “HydABC”-type family of electron bifurcating enzymes as BfuABC where Bfu refers to their predicted ability to reversibly bifurcate electrons to NAD^+^ and Fd, and about half of the family members use various compounds rather than H_2_ as the third substrate.

**FIGURE 2:**
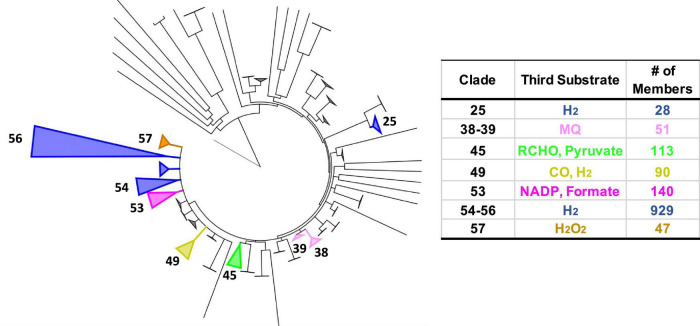
Phylogenetic tree of Bfu enzymes based on BfuB and distribution of characterized and predicted functions as defined by the third substrate. **Left:** tree based on 1558 BfuB homologs (containing InterPro domains IPR011538, IPR019554, IPR019575, and IPR017896). BfuB clades were defined as having a branch root length of <0.33 to minimize the number of clades while highlighting functional differences, leading to 57 total clades (23 orphan clades). Clades with predicted functions are numbered. **Right:** number (#) of different enzymes in each clade classified by the third substrate utilized [in addition to Fd and NAD(H)]. Clade sizes are proportional to the number of members. The non-bifurcating BfuB homolog Nqo1 from *Thermus thermophilus* was used as the outgroup.

**FIGURE 3:**
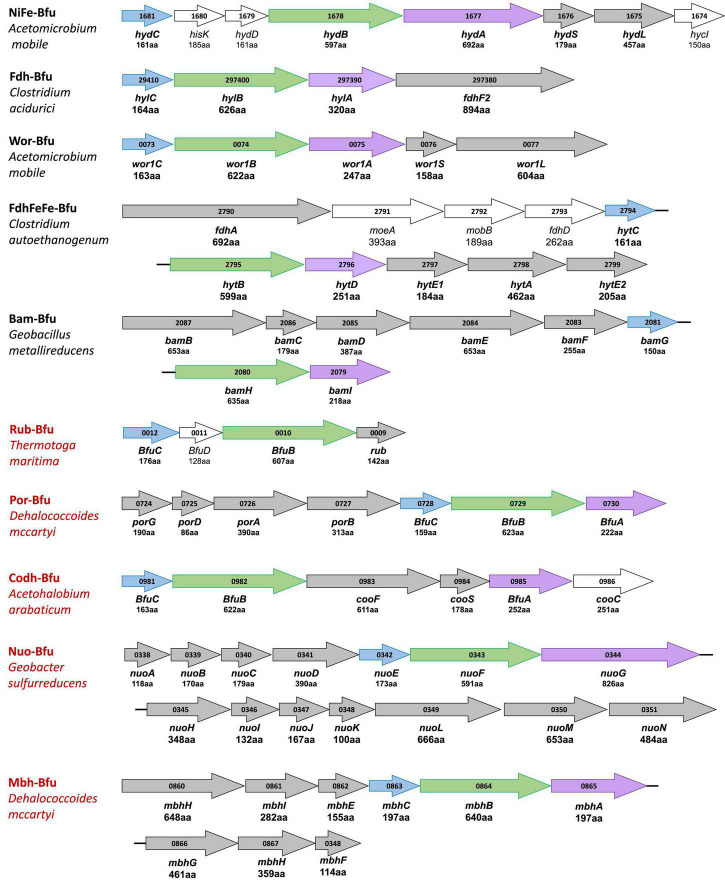
Gene cluster organization of characterized and predicted electron bifurcating complexes with homologous Bfu(Hyd)ABC domains. Characterized and predicted enzymes and their microbial sources are shown in black and red font, respectively. Homologous Bfu subunits are colored purple (BfuA), green (BfuB), and blue (BfuC) where the colors correspond to those used in Figures 1, 6–9 and Supplementary Figures 1A–F. Additional subunits of the Bfu enzyme are shown in gray while genes encoding processing or regulatory proteins are designated by plain text and are not colored. Numbers inside the arrows refer to the gene number. The numbers of amino acids that are encoded by each gene are listed under the gene name. Bold gene names are part of the holoenzyme.

The extended HydABC-like family, now referred to as the BfuABC family, is defined herein by specific InterpPro domains in BfuB (IPR011538, IPR019554, IPR019575, and IPR017896) and in BfuC (IPR002023). The diverse collection of BfuA homologs that we identified contain at a minimum domains IPR017896 and IPR001041. A phylogenetic tree of the 1558 BfuB sequences reveals 57 distinct clades, 9 of which have more than 20 members and 23 of the 57 are orphan (Figure 2). The BfuB sequences associated with FeFe-hydrogenases (previously HydB) are mainly within BfuB clades 54 and 56 (928 members) and those of the NiFe-hydrogenases mainly in clade 49 (6 members; Supplementary Figures 1, 2). Remarkably, there are a large number of other functions encoded by additional genes that are part of the other BfuABC-like clusters. As shown in Figure 3, these include genes encoding pyruvate oxidoreductases [Por, 12 sequences (Charon et al., 1999)], carbon monoxide dehydrogenases [Codh, 4 sequences (Jeoung and Dobbek, 2007)], F_420_ dehydrogenase [F_420_, 2 sequences (Grinter and Greening, 2021)], tungstopterin-containing oxidoreductases [Wor, 37 sequences (Nissen and Basen, 2019)], aryl-CoA thioester reductases [Bam, 19 sequences (Huwiler et al., 2019)], rubrerythrins [Rub, 13 sequences (Nogueira et al., 2021)], and formate dehydrogenases [Fdh, 82 sequences (Radon et al., 2020)]. In addition, as shown in Figure 3, there are two BfuABC-containing gene clusters that are predicted to encode membrane bound respiratory complexes that pump protons, NiFe-hydrogenases [Mbh, 10 sequences, group 4 (Schoelmerich and Muller, 2020)] and Complex I-like quinone oxidoreductases [Nuo, 51 sequences (Baradaran et al., 2013)].

**FIGURE 4:**
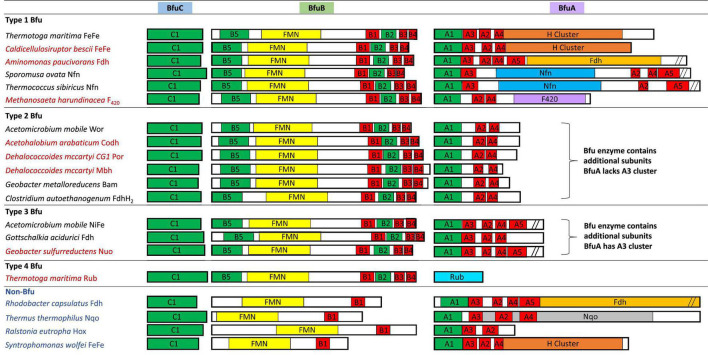
Redox cofactor contents of the BfuABC subunits in the four types of characterized and predicted enzyme in the BfuABC family. Characterized and predicted enzymes and their microbial sources are shown in black and red font, respectively. The cofactors in homologous subunits in non-bifurcating non-Bfu enzymes and their sources are shown in blue font. The cofactors in the BfuBC subunits are conserved in all Bfu complexes but not in homologous non-Bfu enzymes. [2Fe-2S] clusters are indicated by green boxes, [4Fe-4S] by red boxes, FMN by yellow boxes, and additional domains such as those of the H-cluster, pyranopterin, Nfn-like, F_420_ dehydrogenase, rubrerythrin, and Nqo are indicated by brown, orange, blue, purple, light blue, and gray boxes, respectively.

**FIGURE 5:**
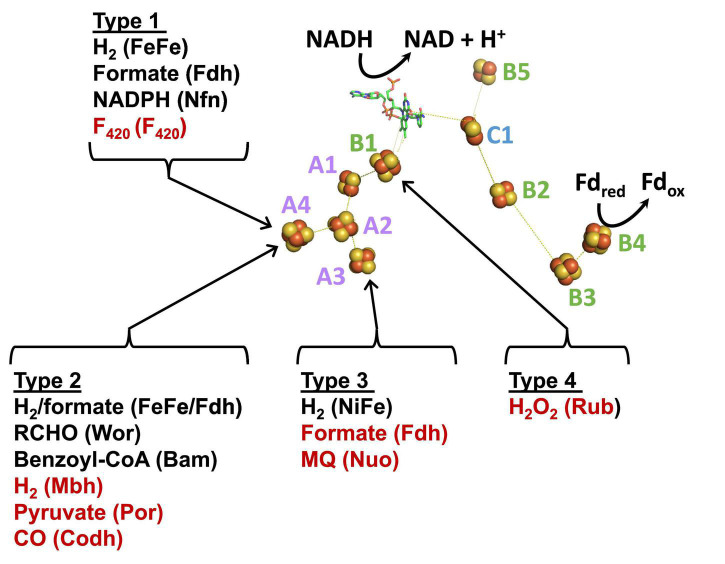
A model of the minimum cofactor content and the predicted electron transfer pathways in characterized and uncharacterized Bfu enzymes. Characterized and predicted enzymes and their microbial sources are shown in black and red font, respectively. Electron donor sites for the Type 1–Type 4 Bfu enzymes in the BfuA subunit are based on the cofactor contents of the A subunits (see Figure 4). The cofactors in the BfuB and BfuC subunits are conserved in all Bfu complexes while there is variation in the cluster content in BfuA although all contain the A1 and A2 clusters. See text for abbreviations.

**FIGURE 6:**
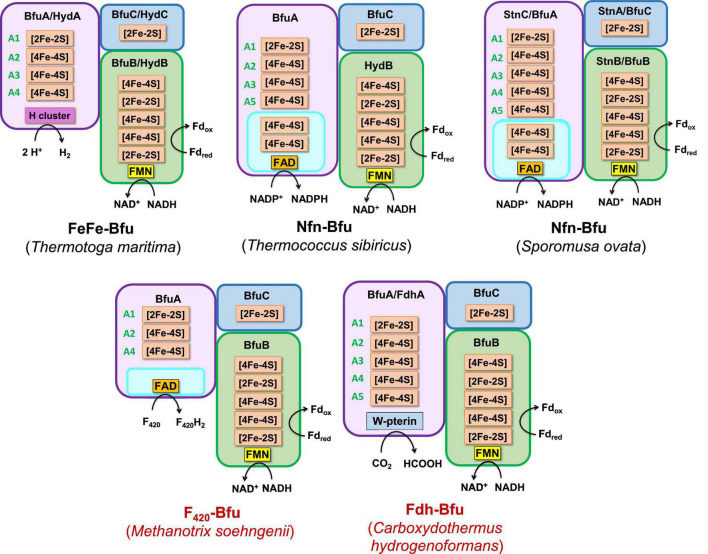
Predicted subunits and cofactor contents of Type 1 Bfu enzymes. Characterized and predicted enzymes and their microbial sources are shown in black and red font, respectively. Each of the BfuA subunits of these enzymes contains an additional domain that enables the enzyme to interact with the third substrate [in addition to Fd and NAD(H)]. See text for details.

**FIGURE 7:**
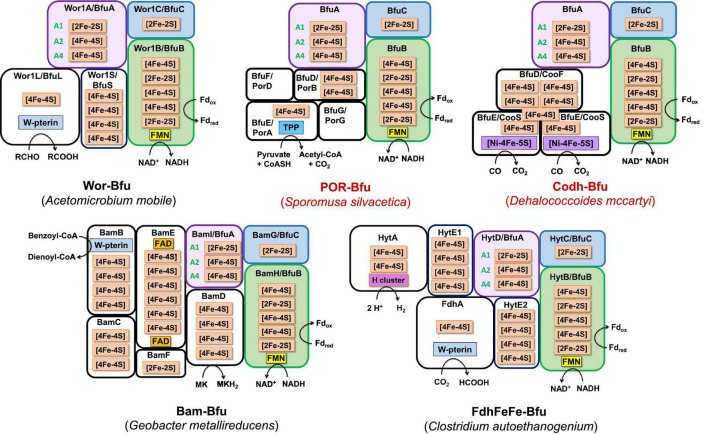
Predicted subunits and cofactor contents of Type 2 Bfu enzymes. Characterized and predicted enzymes and their microbial sources are shown in black and red font, respectively. These enzymes contain additional subunits that feed electrons to BfuA from the third substrate. Their BfuA subunits lack the A3 and A5 iron sulfur clusters. See text for details.

**FIGURE 8:**
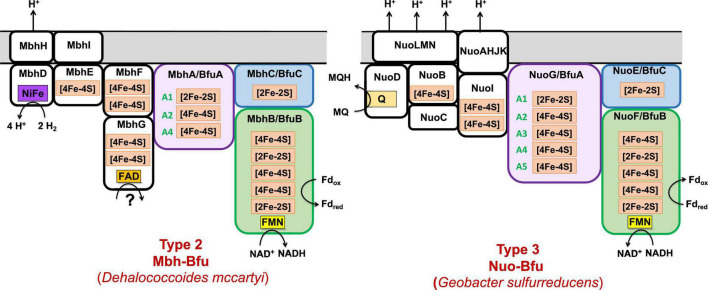
Predicted subunits and cofactor contents of uncharacterized membrane-bound Bfu enzymes. See text for details.

**FIGURE 9:**
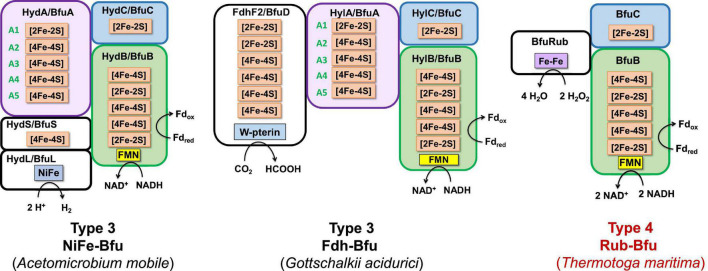
Predicted subunits and cofactor contents of Type 3 and Type 4 Bfu enzymes. Characterized and predicted enzymes and their microbial sources are shown in black and red font, respectively. The Type 3 enzymes contain additional subunits that feed electrons to BfuA from the third substrate and their BfuA subunits contain the A3 and A5 iron sulfur clusters. The Type 4 enzyme lack a BfuA homolog. See text for details.

Each of the BfuABC gene clusters that are hypothesized to encode the different functionalities are associated with phylogenetically distinct clades of BfuB subunit (Figure 2). For example, BfuB clade 45 contains sub-clades for Por, Wor, Bam, Mbh, F_420_, and dual function FdhFeFe (Supplementary Figure 4), while BfuB clade 49 is composed of individual sub-clades that contain either NiFe, Codh, Fdh, or FeFe functionalities (Supplementary Figure 3), and clade 53 is dominated by multiple sub-clades containing Nfn, Fdh, and FeFe sub-clades (Supplementary Figure 5). Many of the functionalities of the various BfuBs are associated with specific types of microorganism. For example, in clade 45, Wor (Synergistetes and Firmicutes), FdhFeFe (Firmicutes), Bam (Proteobacteria) and Mbh and Por (both Chloroflexi) functionalities are all associated with the indicated phylum (Supplementary Figure 4). The same is true for the NiFe (Synergistetes), Fdh (Firmicutes), and FeFe (Synergistetes and Firmicutes) functionalities in clade 49 (Supplementary Figure 3). The BfuB subunits associated with Nfn in clade 53, however, are more microbially diverse as they are found predominantly in Euryarchaeota and Firmicutes, but also in Synergistetes and Bacteroidetes, while in that same clade Fdh is found in Firmicutes and Proteobacteria (Supplementary Figure 5). Additionally, clades 38 and 39 are both made up mainly of Nuo, but are divided between Deferribacteres and Proteobacteria (clade 38) and Planctomycetes and Nitrospirae (clade 39) (Supplementary Figure 6). For 17 BfuB clades (1 through 17), it is not obvious what functions they should be assigned, indicating that a much larger range of metabolic capabilities and diversity exists for the electron bifurcating Bfu family that could be discovered in future studies (Figure 2). In total, we have identified six new predicted functionalities of previously unknown electron bifurcating enzymes, one of which is characterized in this work as described below, and have significantly expanded the size of the families of the already characterized BfuABC-type complexes.

We, therefore, hypothesize that all of these newly predicted BfuABC-type enzymes carry out reversible electron bifurcation reactions using, just like the bifurcating NiFe- and FeFe-hydrogenases, Fd and NADH as two of their three substrates, but they use a substrate other than H_2_ as the third. In addition, we propose that the BfuABC family be classified by the functionality of BfuA, either by the addition of a catalytic domain to BfuA itself or by the presence of additional subunits that feed electrons to and from BfuA. Although the nomenclature for these Bfu enzymes is complicated by the fact that some members of this diverse family already have well established names and assigned genes, herein we propose that they all come under the BfuABC umbrella. Hence, the well characterized FeFe-hydrogenases encoded by HydABC will now be referred to FeFe-BfuABC enzymes, or FeFe-Bfu in an abbreviated form, and NiFe-HydABCSL is abbreviated as NiFe-Bfu. Similarly, as discussed below, the Bfu family also contains members with Por-, Codh-, Bam-, F_420_-, Wor-, Mbh-, Nuo-, Rub-, Nfn-, Fdh-, and FdhFeFe-based functionalities, either by modification of the BfuA subunit or by the presence of additional subunits (Figure 4).

## Classification of BfuABC Enzymes

Our proposal for a large and diverse electron bifurcating Bfu family is supported by the properties of the known characterized members that have been shown to catalyze an electron bifurcating reaction that utilizes Fd, NAD(H) and a third substrate that is not H_2_, although not previously considered to be part of a large diverse family of electron bifurcating enzymes. These are the formate-oxidizing Fdh from *Gottschalkia acidurici* (Wang et al., 2013b) and *Clostridium autoethanogenum* (Wang et al., 2013a), NADP-reducing NADH Fd oxidoreductase (Nfn) from *Sporomusa ovata* (Kremp et al., 2020) and the tungsten-containing aldehyde oxidoreductase (Wor) from *A. mobile* (Schut et al., 2021). In fact, the *C. autoethanogenium* enzyme is bifunctional that, as discussed below, also contains an H_2_-oxidizing H cluster domain of an FeFe-hydrogenase but it is not in BfuA. These enzymes contain an absolutely conserved BfuBC core in terms of predicted cofactor content (Figure 4) but vary in how BfuA has been modified. Hence, Nfn-Bfu has a modified BfuA that binds NADP(H), while the Fdh- and Wor-Bfu enzymes contain additional subunits, as discussed below. In contrast, there are several enzymes that have been characterized that contain homologs of the BfuABC subunits but they do not bifurcate. These include *Rhodobacter capsulatus* Fdh, *Thermus thermophilus* NADH quinone oxidoreductase (Nqo), *Ralstonia eutropha* NiFe-hydrogenase and *Syntrophomonas wolfei* FeFe-hydrogenase (Fritsch et al., 2011; Baradaran et al., 2013; Losey et al., 2017; Radon et al., 2020). As shown in Figure 4, the BfuB homologs in these enzymes lack the B2–B5 iron sulfur clusters found in all of the true electron bifurcating Bfu homologs. Interestingly, the B1 cluster found in these non-bifurcating enzymes enables electron transfer between their subunits homologous to “BfuB” to the “BfuA” subunit. Consistent with this, these non-bifurcating homologs also bind and oxidize NADH but they do not interact with Fd.

The proposed Bfu family members all contain an invariant electron bifurcating BfuBC core that contains six conserved iron sulfur clusters (five in BfuB and one in BfuC) and they can be classified into one of four types based on the nature of the BfuA subunit (Figure 4). With Type 1 Bfu enzymes, the BfuA subunit has been itself modified to include a functional catalytic domain (such as the H cluster domain in BfuA of the FeFe-hydrogenases) that interacts with the third substrate, while Type 2 and Type 3 Bfu enzymes contain additional subunits (such as the BfuSL subunits in the NiFe-ABCSL hydrogenase) that interact with the third substrate and feed electrons into the BfuA subunit. Types 2 and 3 are distinguished by which of the iron sulfur clusters in BfuA are used to transport electrons from the third substrate to the BfuBC core. In addition, albeit limited in number, there is a fourth variety of Bfu enzyme, Type 4, where a BfuA homolog is not present and unrelated subunits interact with the third substrate and feed electrons directly into the electron bifurcating BfuBC core. We will now describe each of the four types in detail.

Type 1 Bfu enzymes are represented by the prototypical FeFe-Bfu hydrogenase, where its BfuA contains the H_2_-producing H cluster-containing domain that feeds electrons to the BfuBC core through the A4 cluster (Figure 5). In the other Type 1 enzymes, the BfuA subunit has been modified by the presence of another catalytic domain in place of the H cluster and this is also proposed to feed electrons to BfuBC *via* the A4 cluster (Figure 5). As shown in Figure 6, these include a NADP^+^-reducing, FAD-containing domain homologous to that found in the large subunit of NfnAB (Lubner et al., 2017) in Nfn-Bfu (clade 53), a formate-oxidizing pterin cofactor-containing domain in Fdh-Bfu (clade 49) and a cofactor F_420_-reducing FAD-containing domain in F_420_-Bfu (clade 45). Of these, only the bacterial Nfn-Bfu has been characterized, from *S. ovata*. This enzyme is proposed to link the NAD(P)^+^ and Fd redox carrier pools in this acetogenic bacterium (Wang et al., 2013b; Kremp et al., 2020). Most predicted Bfu complexes are within the domain of Bacteria (Supplementary Table 1) but operons encoding Type 1 Nfn-Bfu, Fdh-Bfu, and F_420_-Bfu are found in some Archaea, although until now none have been characterized. Herein, we have purified the first archaeal member of the Bfu family, the Type 1 Bfu-Nfn enzyme that we predicted was encoded in the genome of *Thermococcus sibiricus* (Tsib). This is of some significance because, surprising, an FeFe-HydA(BC) hydrogenase has yet to be found in a member of the Archaea domain even though such enzymes are widespread in anaerobic bacteria (Poudel et al., 2016). This raises the question can Archaea synthesize all cofactors of a HydA(BC)-type enzyme. Our characterization herein of a Bfu-Nfn enzyme suggests that it is the novel iron–sulfur-containing H cluster of the FeFe-enzymes that has yet to be synthesized by an Archaeon.

*Thermococcus sibiricus* is a member of the Euryarchaeota and grows up to 88°C by peptide fermentation (Miroshnichenko et al., 2001). We expressed the operon encoding Tsib Nfn-BfuABC in the archaeon *Pyrococcus furiosus*, which grows optimally at 100°C (Fiala and Stetter, 1986). Tsib Nfn-Bfu is similar to the Nfn-Bfu from the bacterium *S. ovata* (Kremp et al., 2020), but that enzyme has a much larger BfuA (by ∼200 residues) and contains the A1–A5 clusters (Figure 6). In contrast, the Tsib enzyme lacks the cysteines coordinating the A4 cluster and must therefore feed electrons to BfuBC *via* the A3 cluster (Figure 5). As shown in Supplementary Figure 7, Tsib Bfu-Nfn catalyzes the tightly coupled NADPH-dependent reduction of Fd but only when NAD^+^ is added, confirming our prediction that this a genuine Nfn-Bfu. We also predicted that the FAD in Tsib Nfn-BfuA provides a binding site for NADP(H) and is not a bifurcation site because it seems more likely that the NADP^+^-interacting FAD connects to the iron sulfur clusters in BfuA and forms the electron pathway to the BfuBC core (Supplementary Figure 7). Nfn-Bfu forms the majority of the members of BfuB clade 53 and the archaeal (in the Euryarchaeota) and the bacterial (mainly in the Firmicutes) enzymes are phylogenetically distinct (Supplementary Figure 5). Interestingly, within members of the archaeal family of Thermococcaceae, either Nfn-Bfu or the unrelated bifurcating NfnAB (Lubner et al., 2017) are present in each organism but not both. The other Type 1 archaeal enzyme, F_420_-Bfu, is not found in bacteria and is present in the genomes of some acetoclastic methanogenic archaea of the Methanosaetaceae family (Supplementary Table 1). These organisms are thought to utilize F_420_ for biosynthesis but do not contain genes encoding the traditional F_420_ dehydrogenases (Welte and Deppenmeier, 2014; Grinter and Greening, 2021). We propose that F_420_-Bfu oxidizes NADH and Fd to generate reduced F_420_ for biosynthetic reactions analogous to the Nfn-Bfu reaction that generates NADPH for biosynthesis.

Type 2 enzymes are much more complex than those of Type 1 as they contain additional subunits that are proposed to feed electrons from the third substrate into the BfuA subunit, analogous to the situation with the NiFe-hydrogenase (Figures 3, 7). Their BfuA subunits by definition lack the A3 and A5 clusters (Figure 4) and so they must all feed electrons to the BfuBC core *via* their A4 cluster (Figure 5). Three Type 2 Bfu enzymes have been characterized. The prototypical and least complex member is the tungsten-containing Wor-Bfu (BfuABCSL) from *A. mobile*, where the SL subunits are homologs of aldehyde oxidoreductase (Schut et al., 2021). It represents a small Wor1-Bfu group found in Synergistetes but a much larger Wor-Bfu group in the Firmicutes is also found in BfuB clade 45 (Supplementary Figure 4 and Supplementary Table 1). Clade 45 contains a sub-clade of what we call the bifunctional FdhFeFe-Bfu. In addition to the expected BfuABC subunits, it contains additional subunits containing a pterin site in FdhA and an H cluster for FeFe-hydrogenase activity in the HytA subunit, while the HytD/BfuA subunit only contains the A1, A2, and A4 clusters (Figure 7). In *C. autoethanogenum*, it was shown that this FdhFeFe-Bfu complex can reversibly generate either H_2_ or formate (from CO_2_) using Fd and NADH as electron donors, as well as interconvert H_2_/CO_2_ and formate (Wang et al., 2013a).

The Type 2 family also includes one of the most complex of the Bfu enzymes, Bam-Bfu, which has been purified and a subcomplex (BamBC) was structurally characterized (Weinert et al., 2015). The holoenzyme contains eight subunits, designated Bam-BfuBCDEFGHI, where the I, H, and G subunits are BfuABC homologs, respectively (Figures 3, 7). These complexes have been characterized from *Geobacter* and *Desulfosarcina* species and are involved in the anaerobic degradation of aromatic compounds by the low potential reduction of benzoyl-CoA (Anselmann et al., 2019; Huwiler et al., 2019). Bam-Bfu complexes also contain a HdrA-type electron bifurcating module and a tungstopterin domain that is the site where the benzoyl-CoA is reduced. It was proposed that, through an unknown electron bifurcating mechanism, low potential electrons are generated to overcome the limiting step in benzoate degradation, the aromatic ring reduction of benzoyl-CoA (Fuchs et al., 2011). Although extensive research has been performed with these complex enzymes, the true nature of the electron bifurcation reaction could not be demonstrated (Huwiler et al., 2019). Based on our analysis here, we propose that the BfuABC (BamGHI) module bifurcates NADH and Fd to supply mid potential electrons (E′∼−400 mV) to a second electron bifurcating module, HdrA, that then generates a low potential electron for benzoyl-CoA reduction (E°′ = −622 mV) and a high potential electron, which presumably is ultimately transferred to the quinone pool (Huwiler et al., 2019). Hence, the BfuABC module of Bam-Bfu is proposed to oxidize Fd_red_ and NADH but is not directly involved in substrate (benzoyl-CoA) reduction, thereby demonstrating the versatility of this module in energy-requiring electron transfer reactions.

There are numerous Type 2 Bfu enzymes that have yet to be characterized. They include Codh-Bfu (A-F), which is proposed to use CO as the third substrate where three subunits in addition to BfuABC contain the Ni-Fe-S active site and multiple [4Fe-4S] clusters (Figure 7). Of course, this raises the fundamental question of whether the Codh-related genes within the gene cluster that also encodes the BfuABC subunits are truly encoding additional subunits of a Codh-Bfu enzyme, or simply a standalone CODH enzyme whose operon happens to be adjacent to that encoding BfuABC. The latter would seem highly unlikely since the genes encoding the Codh enzymes in both *Acetohalobium arabaticum* and *Clostridium cellulovorans* are sandwiched between the BfuB and BfuA genes (Figure 3). Codh-Bfu is found in clade 49 and only in Firmicutes (Supplementary Figure 3 and Supplementary Table 1) and presumably oxidizes CO for energy generation and/or as a detoxification system. Two other uncharacterized Bfu enzymes, Por- and Mbh-Bfu (Figure 3), are found as distinct sub-clades of BfuB clade 45 (Supplementary Figure 4). Por-Bfu (A-G) has four pyruvate oxidoreductase subunits (DEFG) with a thiamine pyrophosphate binding catalytic site and additional [4Fe-4S] clusters in Por-BfuE (Figure 7). Support for an operon encoding a single Por-Bfu enzyme (Figure 3) rather than two distinct enzymes (POR and BfuABC) comes from a whole genome transcript analysis of *Dehalococcoides mccartyi 195* at different growth phases (Johnson et al., 2008). The genes encoding Por-Bfu, which are sandwiched between two tRNA genes, all show similar expression levels during all growth phases tested. The Por-Bfu gene cluster is mainly found in organohalide-respiring Chloroflexi and syntrophic Proteobacteria species, where it could be used to generate pyruvate for gluconeogenesis using NADH and Fd as electron donors, presumably under energy limitation (clade 35, 44, and 45, Supplementary Table 1).

The organohalide-respiring Chloroflexi such as *Dehalococcoides* species are also predicted to contain an uncharacterized membrane-bound NiFe-hydrogenase linked to a BfuABC domain (Mbh-Bfu, clade 45) that also appears to translocate protons (Figure 8). The gene cluster encoding it contains six genes homologous to those encoding an energy converting NiFe-hydrogenase (Schoelmerich and Muller, 2020) that are surrounding those encoding the BfuABC subunits (Figure 3). It would seem extremely unlikely that this gene cluster encodes two distinct enzymes (Mbh and BfuABC) given this gene arrangement. Support for a 9-gene operon encoding an Mbh-Bfu enzyme also comes from transcript analysis of *D. mccartyi 195* mentioned above in which the Mbh-Bfu genes are expressed at comparable levels (Johnson et al., 2008). Interestingly, one of the nine subunits (Mbh-BfuG) has an additional flavin-binding site of unknown function suggesting that this enzyme has a complex and as yet unknown function (Figure 8). This membrane-bound Mbh-Bfu complex likely oxidizes H_2_ to produce reduced Fd and NADH for reductive dehalogenation and biosynthesis as these *Dehalococcoides* species use H_2_ as an electron source for organohalide respiration (Hartwig et al., 2017). From a phylogenetic perspective, all members of BfuB clade 45 (including F_420_-, Wor-, Bam-, and FdhH_2_-Bfu) contain only the A1, A2, and A4 [4Fe-4S] clusters in their BfuA subunit and feed electrons to the BfuBC core *via* the A4 cluster (Supplementary Figure 4).

Type 3 Bfu enzymes also contain subunits in addition to BfuABC but, in contrast to the Type 2 enzymes, contain the A3 cluster in BfuA. Type 3 enzymes are represented by the prototypical and structurally characterized *A. mobile* NiFe-Bfu (Figures 1, 9; Feng et al., 2022). This feeds electrons to the BfuBC core *via* the A3 cluster and the same is assumed to be true for the other Type 3 enzymes (Figure 5) even though they also contain the A4 cluster (Figures 8, 9). The NiFe-Bfu hydrogenase is part of a small sub-clade of Bfu complexes in BfuB clade 49 found in the Synergistetes (Supplementary Figure 3). There are archaeal versions of the NiFe-Bfu enzyme in clade 19 that are represented by anaerobic *Halorhabdus* species (Antunes et al., 2008). Other examples of Type 3 enzymes include the Fdh-Bfu of *G. acidurici* that is represented as a single branch in BfuB clade 56 (Supplementary Figure 1). It contains a BfuA subunit with cluster A1–A5, but the pyranopterin site is located in an additional Fdh subunit (Figure 9). This tungsten- and selenium-containing enzyme was purified and shown to bifurcate electrons from formate to NADH and Fd and plays a role in the fermentation of uric acid (Wang et al., 2013b).

The Type 3 enzymes also include a second example of a predicted membrane-bound, proton-translocating Bfu enzyme and this is the 14-subunit Nuo-Bfu (A-N) in BfuB clades 38 (Deferribacteres and Proteobacteria) and 39 (Planctomycetes and Nitrospirae; Supplementary Figure 6). Like Mbh-Bfu, the genes encoding the BfuABC subunits in Nuo-Bfu sit in the middle of the gene cluster (Figure 3), consistent with this being an operon encoding the 14 subunits of Nuo-Bfu. Support for this operon comes from both proteomics and transcriptomics analyses of *Geobacter sulfurreducens* when grown on acetate, formate or hydrogen gas as the electron donor, in which all genes of the Nuo-Bfu cluster are clearly co-expressed (Shrestha et al., 2013; Mollaei et al., 2021). Nuo-Bfu resembles Complex I of the aerobic respiratory chain but its BfuABC module replaces the canonical NADH module (NuoEFG) for electron input (Figure 8). Nuo-Bfu is predicted to use menaquinone as the third substrate while the BfuABC module allows proton pumping to be driven by the simultaneous oxidation NADH and Fd_red_. Importantly, a study using *E. coli* respiratory Complex I found that the initial proton pumping rate using menaquinone (E°′ = −74 mV) as the electron acceptor was only half of that with ubiquinone (E°′ = 100 mV), suggesting that the energy released from menaquinone reduction is not enough to pump four protons across the membrane (Nakamaru-Ogiso et al., 2014). In contrast, a preceding electron confurcating reaction *via* BfuBC would be able to supply enough energy in the reaction with menaquinone to pump all four protons. Nuo-Bfu also represents a potential intermediate in the evolution of Fd-dependent Complex I, known as NDH1 in cyanobacteria (Pan et al., 2020), into an NADH-dependent enzyme such as the ubiquinone-linked Complex I and Nuo (Kampjut and Sazanov, 2020; Yu et al., 2021).

Type 4 Bfu enzymes have the distinction of containing a BfuBC core but not a BfuA homolog. This serves to emphasize both the importance of the highly conserved BfuBC core (Figure 4) in electron bifurcation and that the role of BfuA is simply to feed electrons to the core from a third substrate. Apparently, that function can also be achieved by completely unrelated subunits. The BfuA-less Bfu enzymes are found in BfuB clade 57, which contains 47 members with only the BfuBC core (Supplementary Figure 8). For many members of this clade, it is not immediately clear from the surrounding genes what the third substrate could be. In addition, for some of them it is possible that a homolog of BfuA is encoded elsewhere in the genome. However, we identified 13 members of clade 57 where an adjacent gene directly downstream of BfuB encodes a homolog of rubrerythrin, a diiron-containing protein that reduces hydrogen peroxide in anaerobes (Nogueira et al., 2021; Supplementary Figure 8). We are designating this predicted complex as Rub-Bfu, a Type 4 Bfu enzyme where the BfuA subunit is completely missing and replaced with an unrelated subunit (Figure 9). We predict that Rub-Bfu family members are capable of using NADH and Fd simultaneously to provide the reductant necessary for detoxification of hydrogen peroxide, even though the redox potential of the hydrogen peroxide/water couple is very high (E°′ = +1349 mV, Supplementary Table 1).

We propose that the Rub-Bfu enzyme is of physiological importance. Most microorganisms, including anaerobes, have a defense system against reactive oxygen species in which reductant is typically supplied by rubredoxin, a monomeric high potential iron-containing redox protein (E_m_ ∼0 mV; Wildschut et al., 2006). The pool of reduced rubredoxin is maintained by a highly active NADPH rubredoxin oxidoreductase (NROR; Ma and Adams, 1999; Nishikawa et al., 2010) and it is assumed that almost all of the rubredoxin in the cell is in the reduced state to efficiently reduce reactive oxygen species ultimately powered by the NADPH pool (Adams et al., 2002; Martins et al., 2019). The physiological redox potential of NADPH in Nfn-Bfu containing anaerobes could also be provided by the NADH/Fd bifurcation reaction of Rub-Bfu, and thus the direct reduction of hydrogen peroxide by the NADH/Fd bifurcating reaction might have distinct physiological advantages for anaerobic organisms exposed to oxidative stress. Hence, coupling the reduction and detoxification of reactive oxygen species to an electron bifurcation reaction with Fd and NADH as the electron donors may be a reasonable detoxification strategy.

While many of the Bfu enzymes described above fall into distinct sub-clades within the BfuB phylogenetic tree (Figure 2), the predicted formate-oxidizing Fdh-Bfu-type enzymes are very distinct as they are distributed throughout the tree. The largest numbers of Fdh enzymes are in BfuB clades 49 and 55 but they are also present in many sub-clades and as individual branches. They are also found in a number of different subunit configurations representing Type 1, Type 2, and Type 3 enzymes (Figures 4, 5 and Supplementary Table 1). The enzyme from *Carboxydothermus hydrogenoformans* is an example of the simplest Type 1 Fdh-Bfu (clade 49, Supplementary Figure 4), in which the BfuA subunit contains a pterin active site for formate oxidation next to the A5 cluster (Figure 6). In Type 2 and 3 Fdh-Bfu enzymes the pyranopterin is located in additional subunits and these are found primarily within the Firmicutes but with some examples in Proteobacteria and Chloroflexi. The Type 2 Fdh includes the bifunctional FdhFeFe-enzyme from *C. autoethanogenium* that lacks the A3 cluster (Figure 7), while the Type 3 Fdh from *G. acidurici* contains the A3 cluster (Figure 9). The ability to bifurcate electrons using formate as the third substrate is clearly an extremely important reaction in anaerobic metabolism, as shown by the widespread distribution and diversity of Fdh-type Bfu enzymes (Supplementary Table 1), suggesting that they evolved multiple times to catalyze the same electron bifurcating reaction based on the BfuBC core.

Our analysis presented herein shows that there is potentially a wide variety of third substrates for the Bfu-type complexes and they represent a paradigm shift in our understanding of electron bifurcation in general. A list of the reactions involving these various third substrates are shown in Table 1, and they fall into three distinct groups, representing low, mid, and high potential reactions. Bfu enzymes that have third substrates involving mid potential reactions (near −400 mV), including H_2_, formate, cofactor F_420_, and NADP(H), are the most widely distributed and best characterized. As pointed out by Buckel and Thauer (2018a), the effective potentials (E′) of NADH and Fd under physiological conditions are −280 and −500 mV, respectively, which is why Fd_red_ readily drives H_2_ production (E°′ = −414 mV) by non-BF hydrogenases but NADH (E′ −280 mV) cannot, except at very low partial pressures of H_2_ (Losey et al., 2017, 2020). Hence the mid potential reactions catalyzed by Bfu-type enzymes nicely fit in energetic terms between NAD(H) and Fd_ox/red_ (Table 1).

**TABLE 1:** Third reactions of Bfu enzymes.

Bfu	Third Reaction	E_*o*_′	^∼^E′
Rub-	H_2_O_2_ + 2H^+^ + 2e^–^ ⇌ 2H_2_O	+1349	
Nuo-	MQH_2_ ⇌ MQ + 2H^+^ +2e^–^	−74	
**NAD/H**	**NAD + 2e^–^ + H^+^ ⇌ NADH**	**−320**	**−280**
Nfn-	NADPH ⇌ NADP + H^+^ + 2e^–^	−320	−380)
F_420_-	F_420_H_2_ ⇌ F_420_ + H^+^ + 2e^–^	−360	
NiFe-/Mbh-	H_2_ ⇌ 2H^+^ + 2e^–^	−414	
FeFe-	H_2_ ⇌ 2H^+^ + 2e^–^	−414	
Fdh-	HCOO^–^ ⇌ CO_2_ + H^+^ + 2e^–^	−420	
**Fd**	**Fd_ox_ + 2e^–^ ⇌ Fd_red_**	**−420**	**−500**
Por-	Pyruvate +CoA ⇌ CO_2_ + Acetyl-CoA + 2e^–^	−500	
Codh-	CO + H_2_O ⇌ CO_2_ +2H^+^ + 2e^–^	−520	
Wor-	RCHO + H_2_O ⇌ RCOO^–^ + 3H^+^ + 2e^–^	−560	
Bam-	Dienoyl-CoA ⇌ Benzoyl-CoA + 2e^–^ + H_2_O	−620	

*The approximate reduction potentials for the Fd_ox/red_, NAD^+^/NADH, and NADP^+^/NADPH redox couples under physiological conditions (E′). High (purple), mid (blue), and low (red) potential reactions are indicated.*

It is not clear why low potential reactions (E^°^′∼−500 mV) involving aldehydes, pyruvate and CO (Table 1) are catalyzed by electron bifurcating Bfu enzymes as the third substrate could reduce Fd and NAD^+^ directly. However, insight into this issue comes from the recently characterized Wor-Bfu from *A. mobile*. This Bfu enzyme was shown to catalyze an electron bifurcating reaction in which it couples aldehyde oxidation (E°′−560 mV) to the simultaneous reduction of NAD and Fd (Schut et al., 2021). Importantly, neither NAD or Fd was reduced in the absence of the other electron acceptor, even though both reactions are exergonic. This strongly supports our hypothesis that in these other Bfu enzymes both pyruvate and CO are oxidized but only when coupled to the simultaneous reduction of NAD^+^ and Fd, and that neither acceptor is reduced in the absence of the other. In the case of Wor-Bfu, we proposed that electron bifurcation enabled a microorganism to oxidize very low concentrations of toxic aldehydes in the human gut even in the presence high concentrations of the corresponding acid, where the effective potential of the RCHO/RCOO^–^ couple would be in the mid potential range and Fd reduction would be endergonic unless coupled to NAD^+^ reduction (Schut et al., 2021). We hypothesize that the same principle applies to Por- and Codh-Bfu enzymes. Hence, the oxidation of CO by a bifurcating Codh-Bfu might enable extremely low concentrations of CO to be utilized.

At the other end of the potential spectrum is Rub-Bfu (Table 1). If this does indeed catalyze the reduction of extremely high potential hydrogen peroxide using Fd and NADH as the electron donors, as discussed above, then the reactions shown in Table 1 would represent a paradigm shift in our understanding of the role of BF-enzymes in microbial metabolism. The range of standard redox potentials of the third substrate would now span almost 2 V, from ∼−500 mV to approaching +1400 mV. Such catalytic flexibility in this class of electron bifurcating enzyme is unprecedented and might explain why the Bfu family is so diverse such that many types of third reaction linked to Fd and NAD(H) reduction or oxidation can be catalyzed.

## A Unifying Electron Bifurcating Mechanism for Bfu Enzymes

**FIGURE 10:**
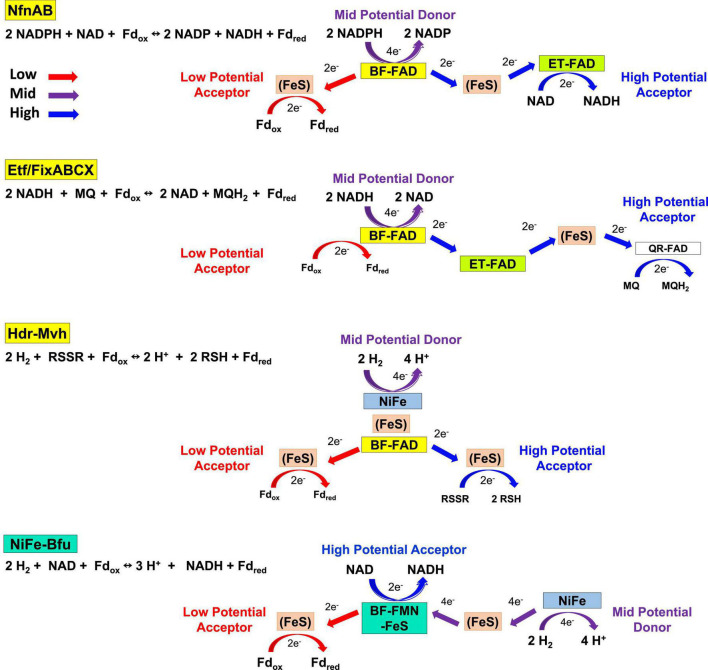
Electron transfer pathways in the four families of electron bifurcating enzymes. The roles of flavin and the electron transfer pathways are depicted in NiFe-Bfu, NfnAB, EtfABCX, and Hdr-Mvh. For simplicity, Fd is depicted as a two-electron carrier. See text for details.

In spite of their diversity in terms of functionality, total subunit composition and the nature of the third substrate, members of the Bfu family all contain a conserved BfuBC core that reversibly reduces NAD^+^ and Fd and we propose that all of these enzymes have the same mechanism of electron bifurcation. This must be different from those of the other three families of BF-enzyme, NfnAB, EtfAB, and HdrA (Buckel and Thauer, 2018a). The pathways of electron flow during the 4 e^–^ catalytic cycles of these other enzymes and of NiFe-Bfu (Eq. 1) are compared in Figure 10 where, in each case, Fd_ox_ is the low potential electron acceptor in the electron bifurcating reaction. In the other BF-enzymes, their BF-FADs accept 2 e^–^ from the mid potential donor [NAD(P)H or H_2_] and bifurcate single e^–^ to high and low potential electron transfer pathways. This process is repeated for the complete 4 e^–^ reaction cycle. In Nfn and EtfABCX, electrons are transferred to the high potential acceptor (NAD or MQ, respectively) *via* a second electron-transferring flavin or ET-FAD. In contrast, in the structurally characterized NiFe-Bfu (Figure 1), all 4 e^–^ from the oxidation of the mid potential donor, H_2_, must pass through the single FMN with the net result of 2 e^–^ (as a hydride) reducing NAD^+^ and 2 single e^–^ reducing Fd_ox_. Hence, the single FMN in NiFe-Bfu appears to function as both a BF- and an ET-flavin at the same time. The Bfu enzymes are, therefore, very distinct from the other three families of electron bifurcating enzymes in terms of the role of the flavin and the pathways of electron transfer between their three substrates. We therefore propose that FMN in the Bfu enzymes, represented by the prototypical NiFe-Bfu, must achieve the bifurcation of 4 e^–^ with the participation of a unique arrangement of nearby iron–sulfur clusters. The electron bifurcation site in this enzyme is therefore designated, not as BF-FAD as in the other BF-enzymes, but as BF-FMN-FeS (Figure 10).

The structure of NiFe-Bfu (Feng et al., 2022) reveals that BfuB contains three [4Fe-4S] clusters (B1, B3, and B4) and two [2Fe-2S] clusters (B2 and B5, Figure 1). While the B2 cluster is typically not predicted in Bfu-family enzymes, biochemical analysis of the *T. maritima* BfuB purified from its native source revealed two [2Fe-2S] clusters based on EPR analysis (Verhagen et al., 1999), in agreement with the second [2Fe-2S] cluster seen in the structure of NiFe-Bfu (Figure 1). In the NiFe-enzyme, the B2 cluster is penta-coordinated with one of the Fe atoms (FeA) liganded by two Cys (C476 and C536) while the other (FeB) is tri-coordinate and coordinated by two Cys (C438 and C531) and one His (H525). The His is conserved in virtually all BfuB sequences (missing in 8 of 1558 sequences) but is not present in HydB homologs that do not bifurcate (Supplementary Figure 9). The B2 cluster was not predicted to be present from sequence analysis of FeFe-hydrogenases as the five coordinating residues span the binding motif of the B1 4Fe-cluster and one of the Cys that coordinate the B2 FeA atom in the NiFe-enzyme, C476, is replaced by Thr in the FeFe-enzymes as well as in about two-thirds of 1558 BfuB sequences (Supplementary Figure 9). Thr coordination is not unusual for [2Fe-2S] clusters (Uhlmann and Bernhardt, 1995) and has the effect of decreasing the cluster redox potential but the His/Cys pentacoordinate coordination seen in the B2 cluster of BfuB is so far unique. Several enzymes contain iron sulfur clusters with non-standard coordination that play novel roles in electron transfer and catalysis, such as the penta- and tri-coordinate Fe atoms in the [4Fe-4S] clusters of Fd thioredoxin reductase (Walters et al., 2005) and sulfane sulfur reductase (Yu et al., 2020), respectively, and we predict that the B2 cluster in BfuB also plays a key role in the electron bifurcation reaction. The BfuC subunit also contains a [2Fe-2S] cluster (C1), and we propose that this B2-FMN-C1 triad plays a special role in electron bifurcation, as discussed below.

**FIGURE 11:**
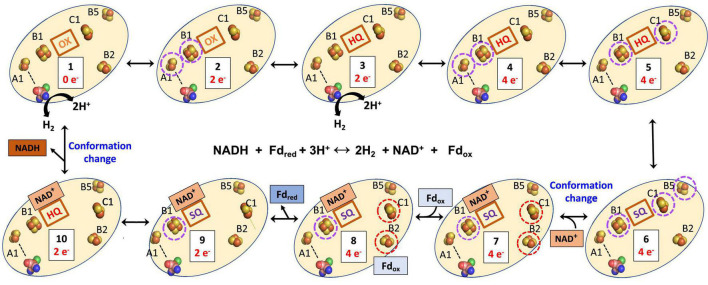
Hypothetical mechanism of electron bifurcation in NiFe-Bfu hydrogenase. The FeS clusters A2, A3, and S1 that link A1 and the NiFe site (see Figure 1) are represented by a dotted line. Not shown are clusters B3 and B4, which are proposed to transfer electrons from B2 to Fd. Low and medium potential redox centers are indicated by red and purple dotted circles, respectively. The FMN is indicated by its redox state as either oxidized (OX), the semiquinone (SQ), or the reduced hydroquinone (HQ). The binding of NADH to the FMN site is predicted to cause a conformational change leading to movement of the C1 cluster away from the flavin toward the B2 cluster. The reverse process occurs when NADH leaves.

The structure of the NiFe-Bfu enzyme (Figure 1) shows that a continuous path of iron sulfur clusters, all within electron transfer range (≤13 Å), lies between the FMN and the NiFe-site providing a path for mid potential electrons from H_2_ in the electron bifurcation reaction. We proposed that electron transfer from the flavin to Fd_ox_ occurs *via* the C1, B2, B3, and B4 clusters (Feng et al., 2022). Based on the bioinformatic data presented herein and the insights provided by the cryo-EM structure, we now put forward a hypothetical mechanism of electron bifurcation for NiFe-Bfu that we propose applies to all members of the Bfu family, regardless of the third substrate (Figure 11). It is based on the conserved core of BfuBC containing the key redox cofactors, clusters B1, B2, and B5 in BfuB and cluster C1 in BfuC (Figure 4), which all are positioned around FMN (Figure 1). In the electron bifurcating reaction catalyzed by the NiFe-enzyme, two molecules of the mid potential donor, H_2_, are oxidized to form NADH and Fd_red_. We propose that the resting enzyme is in the fully oxidized “0 e^–^” form (state 1 in Figure 11) and sequential oxidation of two H_2_ molecules adds four mid potential electrons from the NiFe site that pass *via* the S1, A3, A2, and A1 electron transfer pathway (Figure 1) to the FMN site. The four electrons are distributed among the flavin (SQ) and three clusters (B1, C1, and B5; state 6). However, from the cryoEM structure in the bifurcation ready state (Feng et al., 2022), the distance between the C1 and B2 cluster (19Å) precludes electron transfer, thus precluding a pathway to reduce Fd. Binding of NAD^+^ causes a conformational change (state 7) where movement of the C-terminal domain of BfuC brings the C1 cluster within electron transfer distance (13Å) of the B2 cluster, as shown by structural analysis (Feng et al., 2022). The conformational changes in the structure were seen upon addition of both NADH and FMN, but for the proposed mechanism we assume that NADH binding is the main contributor to these changes. We now hypothesize that the domain movement also generates two low potential electrons on clusters C1 and B2 (state 7), although the mechanism involved and the roles of the flavin and the novel pentacoordinate B2 cluster are not clear at present. Nevertheless, the enzyme is now ready for Fd_ox_ to bind and this is reduced by the low potential electrons *via* the B3 and B4 clusters (state 8) with the release of Fd_red_ (state 9). The subsequent release of NADH (state 10) triggers the conformational change back to the resting state of the enzyme (state 1).

Any mechanism put forward for the NiFe-Bfu enzyme must be fully reversible in nature, in accordance with the catalytic properties of the enzyme (Feng et al., 2022), and that is the case with that proposed here for NiFe-Bfu. Specifically, in the reverse confurcating reaction, binding of NADH to the oxidized enzyme (state 1) reduces the oxidized flavin (OX) by 2 e^–^ to give the hydroquinone (HQ) state (state 10). This is accompanied by a conformational change so that the FMN cannot transfer electrons to the C1 cluster (Figure 11), as demonstrated by the NiFe-Bfu structure (Feng et al., 2022). We propose that the FMN in the NAD(H) bound form is uncrossed, meaning that the HQ/SQ couple is of lower potential than the SQ/OX couple (Yuly et al., 2021). The lower potential HQ/SQ couple can reduce the mid-potential B1 cluster while the high potential SQ/OX couple cannot, resulting in a stable SQ (state 9). Upon binding of Fd_red_ (Eq. 1), the third and fourth electrons are donated sequentially to the B2 cluster (through B4 and B3, state 8). These two low potential electrons are transferred down the chain to the C1 cluster and to the B5 cluster, which we propose is in constant proximity to the C1 cluster and allows two low potential electrons to reside in the C1-B5 unit. The enzyme core now contains the four electrons (two from NADH, two from Fd_red_, Eq. 1) necessary to reduce four protons to generate two H_2_ molecules. We propose that the reduction of the B1-FMN-C1-B5 cofactors (state 7) triggers the release of NAD^+^ from FMN, changing its redox properties and initiating the conformational change in which the C1 cluster (with the reduced B5 still in electron transfer distance) coming in electron transfer distance to FMN and moving away from the B2 cluster. The four electrons in B1-FMN-C1-B5 (state 5) are now connected by the A1–A3 and S1 to the [NiFe] site and electrons can move on by one to [NiFe] to form two molecules of H_2_, regenerating the resting oxidized enzyme (state 1). The FMN cofactor in states 6 to 2 acts as an electron transfer cofactor as two low potential and two high potential electrons are merged to generate four mid potential electrons driven by a protein conformational change to generate state 6.

The essence of our proposed mechanism is that the FMN in BfuB together with the C1, B5, B1, and B2 clusters form a four electron redox potential-regulated “transistor” in which the C1 cluster with its proximal B5 cluster can switch between transferring low potential electrons from/to B2 and transferring mid potential electrons from/to the FMN cofactor. It should be noted that all five of the BfuB clusters, including B5, and the C1 cluster in BfuC, are conserved in all Bfu enzymes (Figure 4). The C1 clusters act as the gate or switch separating the low potential pathway (B2–B4) and the mid potential pathway (FMN, B1, A1–A3, S1). NAD(H) binding forms the check point and drives conformational changes. The reaction is completed by addition of Fd leading to additional NADH and Fd_red_ formation. Hence, in our hypothetical mechanism, it is the binding of NAD(H) that controls the electron bifurcation reaction and, in the absence of Fd, the B2–B4 clusters can be reduced by the C1-B5 clusters but only for one cycle. We propose that the multiple functions that FMN performs are controlled by conformational changes induced by the binding of NAD(H). Indeed, the FMN- and NADH-binding domains of BfuB in the Bfu family of enzymes contain several highly conserved residues (F182, A202, and M204 in *A. mobile* NiFe-Bfu) that are not found in non-bifurcating enzymes (Supplementary Figure 10). From the structure of the NiFe-Bfu enzyme, it is likely that these residues alter the secondary coordination sphere that allows the binding of NAD(H) to FMN and this causes both a change in flavin redox properties and an extensive conformational change.

The Bfu family is therefore unique among the four types of electron bifurcating enzymes now known (Figure 10) in that its single flavin, in combination with a unique arrangement of iron–sulfur clusters, is proposed to perform two different functions during catalytic turnover. In the reversible bifurcation reaction, it donates two electrons as a hydride to the high potential acceptor NAD^+^ but it also accepts electrons from the mid potential pathway formed by the [FeS] clusters in BfuA and transfers them to Fd, the low potential acceptor. This is clearly very different from the role of the BF-FAD in the FBEB enzyme systems based on NfnAB, HdrA, and EtfAB and how a single FMN achieves this is not at all clear based on current knowledge of flavin chemistry. The Bfu family does have one similarity with the HdrA and EtfAB families in that all appear to depend upon significant conformational changes to achieve and control electron bifurcation (Vigil et al., 2021; Watanabe et al., 2021). For example, it has been shown in an EtfAB complex that conformational changes cause the BF-FAD to swap between crossed and uncrossed potentials, which is a key part of the electron bifurcating mechanism (Vigil et al., 2022). Similarly, it was proposed from structural analysis of the Fdh-Hdr-Fmd complex that mid potential electrons reduce the BF-FAD one by one and then, driven by conformational changes, the flavin assumes the bifurcation state in which high and low potential electrons are transferred simultaneously to high and low potential pathways (Watanabe et al., 2021). However, in contrast to the EtfAB and HdrA enzymes, no major structural changes were evident in NfnAB (Lubner et al., 2017).

The proposed unprecedented role of the FMN-FeS center in electron bifurcation in the Bfu enzymes is in sharp contrast to that of BF-FAD in the three types of FBEB enzyme (NfnAB, HdrA, and EtfAB) where it alone generates high and low potential electrons. If the proposed electron bifurcation site is confirmed, it will no longer be accurate to classify members of the Bfu family as FBEB enzymes. Instead Bfu will represent a third class of electron bifurcating family that uses flavin and iron–sulfur clusters to bifurcate electrons, in addition to those that carry out flavin (only)- and quinone-based electron bifurcation. In the future it is anticipated that our hypothetical mechanism (Figure 11) will form the basis for designing spectroscopic analyses along the lines previously used for the NfnAB enzyme (Lubner et al., 2017) to investigate the enigmatic role of FMN and its adjacent iron–sulfur clusters in catalysis by both NiFe-Bfu and the array of other Bfu enzymes that have been identified herein. In addition, site-directed mutagenesis studies of the conserved residues in the FMN- and NADH-binding domains, as well as those coordinating the unique pentacoordinate B2 cluster, should provide valuable insight into the electron bifurcating mechanism, and such studies are underway with the NiFe-Bfu enzyme. This ubiquitous family of Bfu enzymes is clearly capable of a range of metabolic reactions and metabolic functions, and other members of this family have yet to be discovered since 18 of the 57 clades of Bfu enzymes that we identified are as yet uncharacterized (Figure 2).

## Materials and Methods

### Bioinformatic Analyses

In InterPro (version 79), *A. mobile* NiFe-BfuB (previously HydB, Anamo_1678, UniProt ID: I4BYB5_ACEMN) is annotated with the domains IPR011538, IPR019554, IPR019575, and IPR017896, which were entered into the InterPro Domain Architecture search tool and yielded 3400 sequences (Marchler-Bauer et al., 2017). These were filtered to exclude duplicates and metagenomic data, and the resulting 1562 sequences were aligned using Clustal Omega, version 1.2.4, with default parameters (Sievers et al., 2011). The resulting alignment was used to construct a maximum likelihood phylogenetic tree using Geneious Prime version 2019.0.4, and the model tree was refined using 1000 bootstrap replicates. iTOL (version 5.7) was used for analysis and display of the phylogenetic trees (Letunic and Bork, 2016). UniProt Retrieve was used to look up the phylum of the organism for each of the 1562 sequences and label them in iTOL.

### Recombinant Production of *Thermococcus sibiricus* Nfn-Bfu in *Pyrococcus furiosus*

A plasmid for chromosomal expression of the Tsib Nfn-Bfu operon in *P. furiosus* was generated by Gibson assembly (New England Biolabs) and contained the following: the Tsib Nfn-Bfu operon (Tsib1517-1519) under the control of the *P. furiosus* slp promoter (Pslp; consisting of 184 bp directly upstream of the PF1399 gene), an N-terminal 9× His tag with flanking Ala spacer encoded on the 5′ end of Tsib1510, the T1 terminator at the 3′ end of the Tsib Nfn-Bfu operon (5′-aatcttttttag cactttt-3′), 0.5-kb flanking regions targeting homologous recombination at *P. furiosus* genome region 3 (fill in genome region 3 properties), the PgdhpyrF cassette for genetic selection of uracil prototrophy in the auxotrophic background strain *P. furiosus* COM1, and the AprR cassette and pSC101 origin for selection of clones in *Escherichia coli*. The resulting plasmid, termed pDN061, was sequence verified and linearized by restriction digestion prior to transformation of *P. furiosus* COM1 as previously described (Lipscomb et al., 2011). The *P. furiosus* strain containing the genome-integrated Tsib Nfn-Bfu expression cassette was designated MW0668. Fermentation on a 15-L scale of recombinant *P. furiosus* was performed in a custom build fermenter and the cells were grown at 85°C to accommodate production of recombinant *T. sibiricus* Nfn-Bfu. A total of 30 g frozen cells were lysed in 4 volumes of 50 mM phosphate buffer (pH 7.5) with 1 mM dithiothreitol (DTT) and 50 μg/mL DNase I under strict anaerobic conditions in a Coy anaerobic chamber (95% Ar, 5% H_2_). Cell debris and membranes were removed by ultracentrifugation at 100,000 × *g*. Recombinant Nfn-Bfu was captured on a 5-mL HisTrap Crude column (Cytiva) using 50 mM phosphate buffer (pH 7.5) containing 500 mM NaCl and 1 mM DTT. The Nfn-Bfu was eluted with a linear gradient to 1 M imidazole in the same buffer and the enzyme eluted around 100 mM imidazole. Active fractions were pooled and loaded on a 5 mL QHP HiTrap column (Cytiva) and developed with a 0–500 mM gradient of NaCl in 25 mM Tris⋅HCl (pH 8) containing 1 mM cysteine. Active fractions of Nfn-Bfu were checked for purity by size exclusion chromatography (SEC650; Bio-Rad) and gel electrophoresis (Mini-PROTEAN TGX Stain-Free Gels; Bio-Rad). Thirty grams of biomass from 15 L *P. furiosus* culture yielded about 5 mg of *T. sibiricus* Nfn-Bfu.

### Activity Measurements of *Thermococcus sibiricus* Nfn-Bfu

Dye-linked assays were performed in an Agilent Cary 100 UV-vis spectrometer by following the reduction of benzyl viologen at 600 nm (ε = 7400 cm^–1^ M^–1^) at 80°C. The reaction mixture (2.0 mL) contained 50 mM Hepes (pH 7.5), 1 mM benzyl viologen, ∼4 μM sodium dithionite and enzyme, and was started with 0.25 mM NADPH. Activity is reported in units where one unit catalyzes the reduction of 1 μmol of benzyl viologen per minute. Specific activity is calculated using protein estimations based on the Bradford method using bovine serum albumin as the standard. For the bifurcating assay, the reduction of Fd was monitored at 425 nm (ε = 13 mM^–1^ cm^–1^, difference between oxidized and reduced). The reaction mixture (400 μl) contained *P. furiosus* Fd (40 μM), 10 μM FMN, 0.5 mM NADPH. The reaction was initiated by the addition of 1 mM NAD. One unit of Nfn-Bfu bifurcating activity is defined as 1 μmol Fd reduced per minute per milligram.

## Data Availability Statement

The original contributions presented in this study are included in the article/Supplementary Material, further inquiries can be directed to the corresponding author.

## Author Contributions

MA and HL designed the research and edited the manuscript. GS, DH, FP, and XF carried out the research and wrote the manuscript. All authors contributed to the article and approved the submitted version.

## Conflict of Interest

The authors declare that the research was conducted in the absence of any commercial or financial relationships that could be construed as a potential conflict of interest.

## Publisher’s Note

All claims expressed in this article are solely those of the authors and do not necessarily represent those of their affiliated organizations, or those of the publisher, the editors and the reviewers. Any product that may be evaluated in this article, or claim that may be made by its manufacturer, is not guaranteed or endorsed by the publisher.
