# Supplementary material for: An Abundant and Diverse New Family of Electron Bifurcating Enzymes With a Non-canonical Catalytic Mechanism
Source: Front Microbiol. 2022 Jul 8;13:946711. doi: 10.3389/fmicb.2022.946711 (PMC9304861; doi:10.3389/fmicb.2022.946711)
Supplement: Supplementary file 1 [file Data_Sheet_1.pdf]

An Abundant and Diverse New Family of Electron Bifurcating Enzymes With a Non-Canonical Mechanism

Gerrit J. Schut[a*], Dominik K. Haja[a*], Xiang Feng[b], Farris L. Poole[a], Huilin Li[b] and Michael W. W. Adams[a]

[a]Department of Biochemistry and Molecular Biology, University of Georgia, Athens, GA 30602, USA

[b]Department of Structural Biology, Van Andel Institute, Grand Rapids, MI 49503, USA

Supplementary Figures 1-10 and Supplementary Table 1

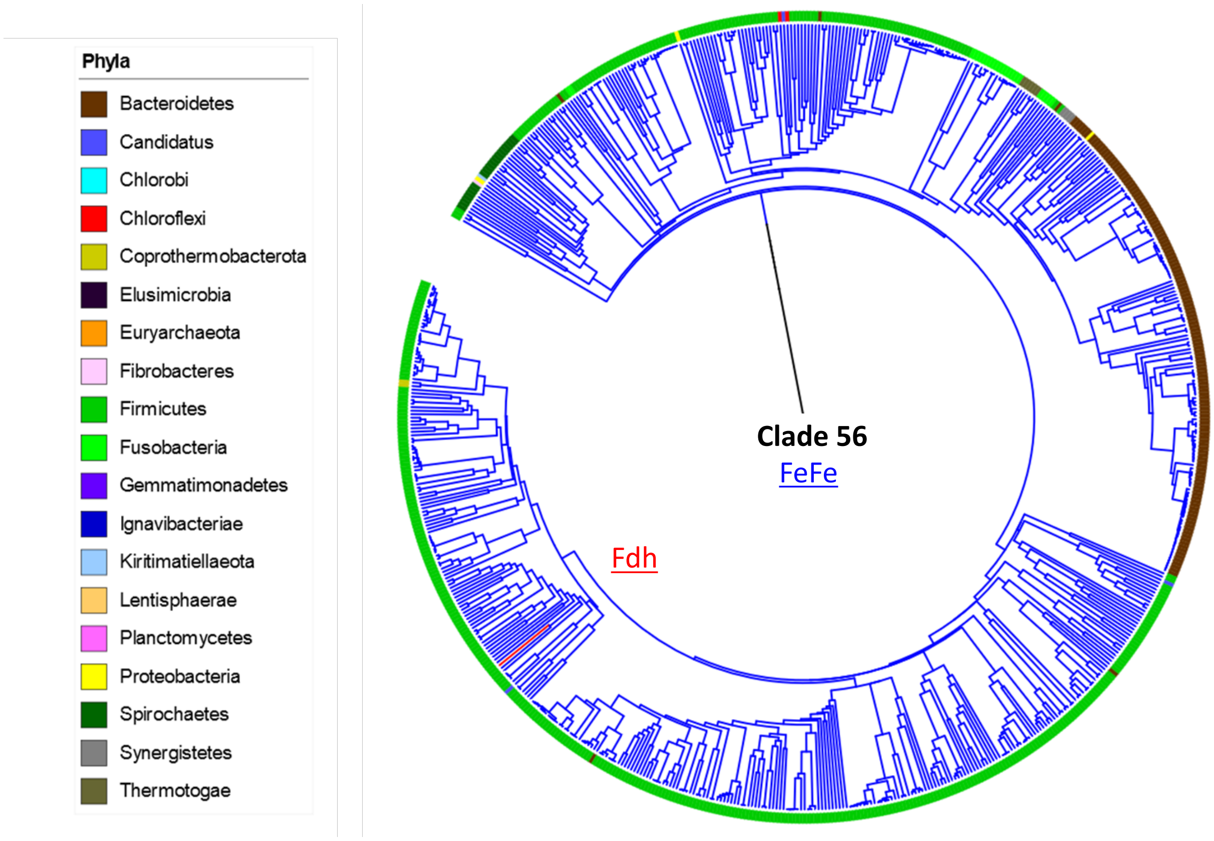

Supplementary Figure 1. Phyla associated with clade 56 of the BfuB phylogenetic tree (see Figure 2). All are FeFe-enzymes (blue) except for a single branch of Fdh (red).

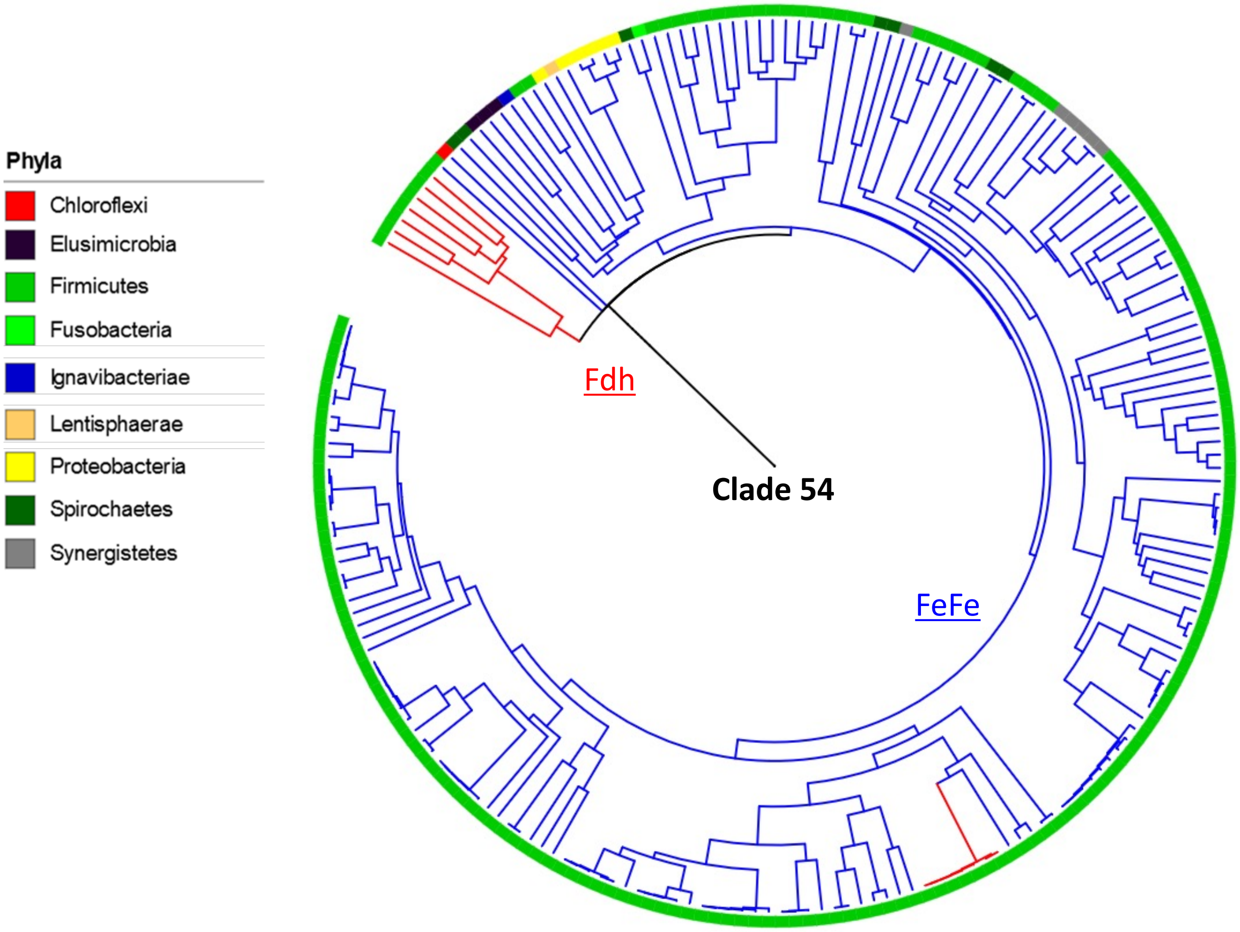

Supplementary Figure 2. Phyla associated with clade 54 of the BfuB phylogenetic tree (see Figure 2). The positions of the Fdh (red) and FeFe (blue) enzymes are indicated.

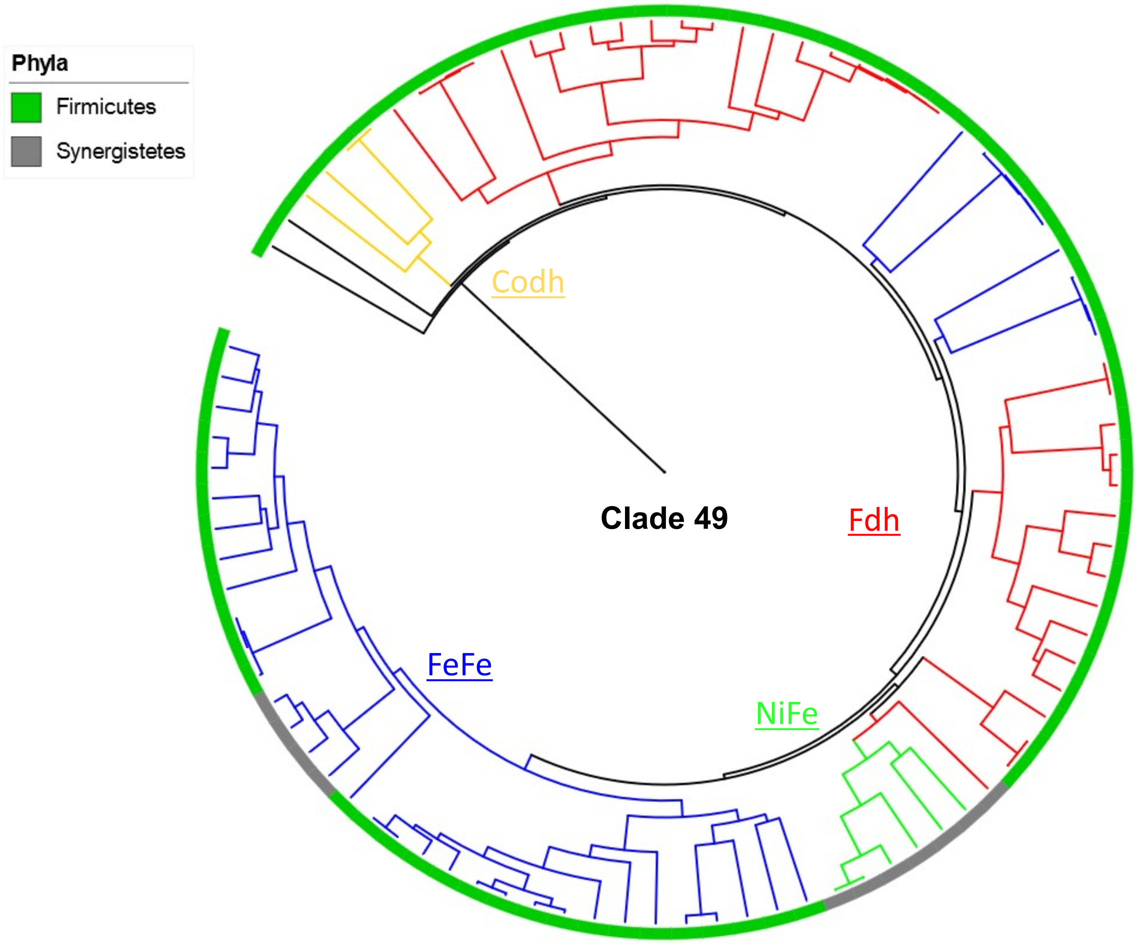

Supplementary Figure 3. Phyla associated with clade 49 of the BfuB phylogenetic tree (see Figure 2). The positions of the Codh (orange), Fdh (red), NiFe (green) and FeFe (blue) enzymes are indicated.

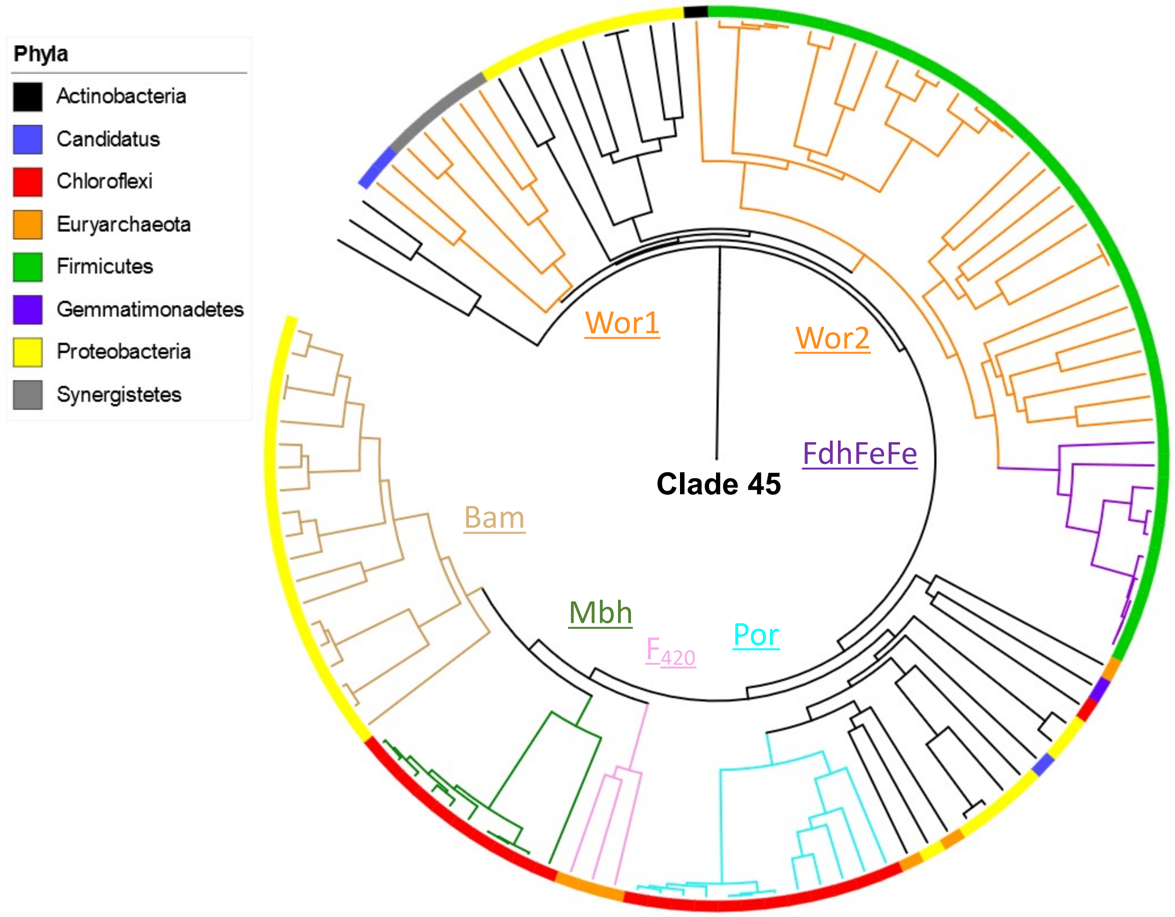

Supplementary Figure 4. Phyla associated with clade 45 of the BfuB phylogenetic tree (see Figure 2). The positions of the Wor1/2 (orange), FdhFeFe (purple), Por (aqua), F_{420} (light purple), Mbh (dark green) and Bam (brown) enzymes are indicated.

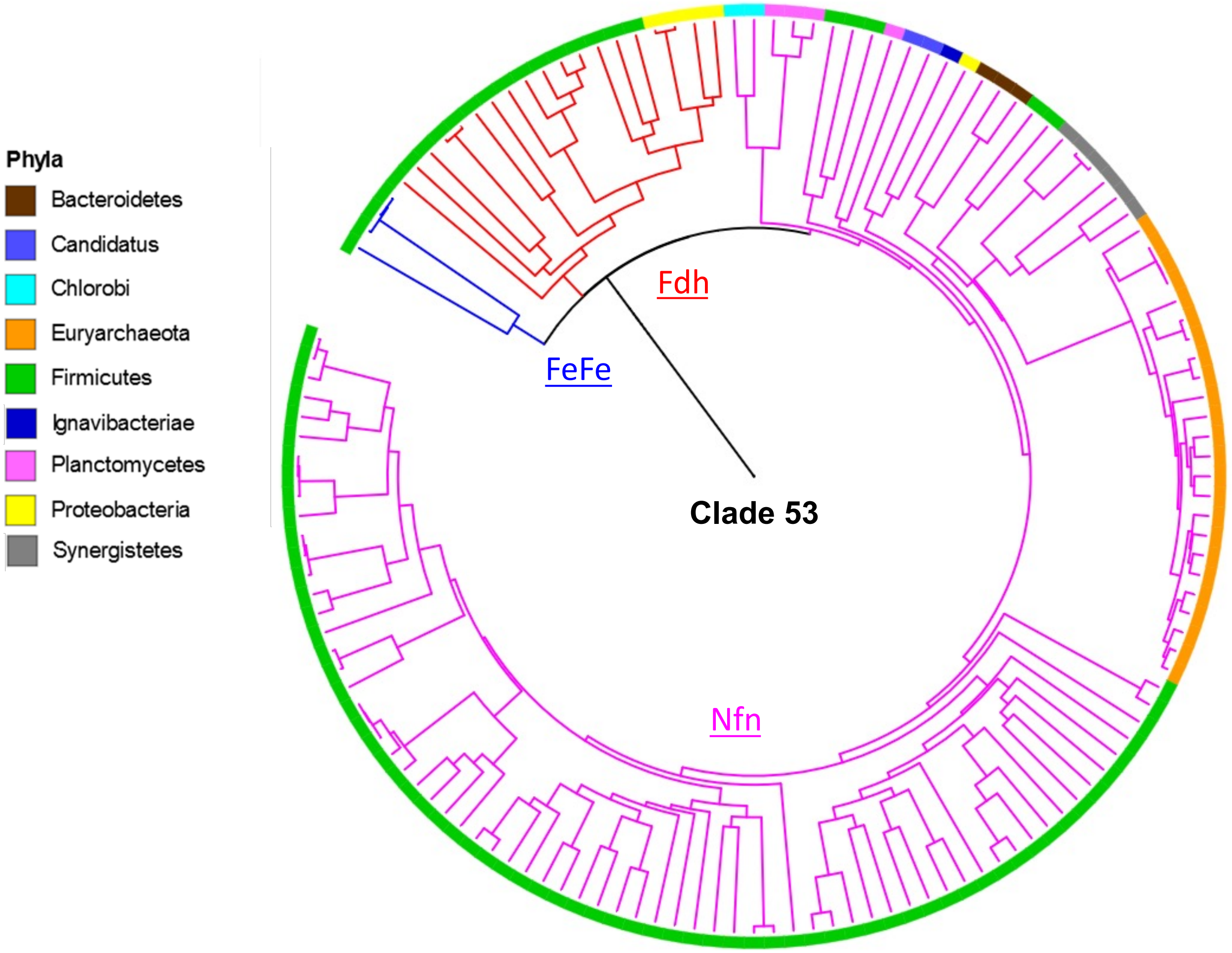

Supplementary Figure 5. Phyla associated with clade 53 of the BfuB phylogenetic tree (see Figure 2). The positions of the FeFe (blue), Fdh (red) and Nfn (pink) enzymes are indicated.

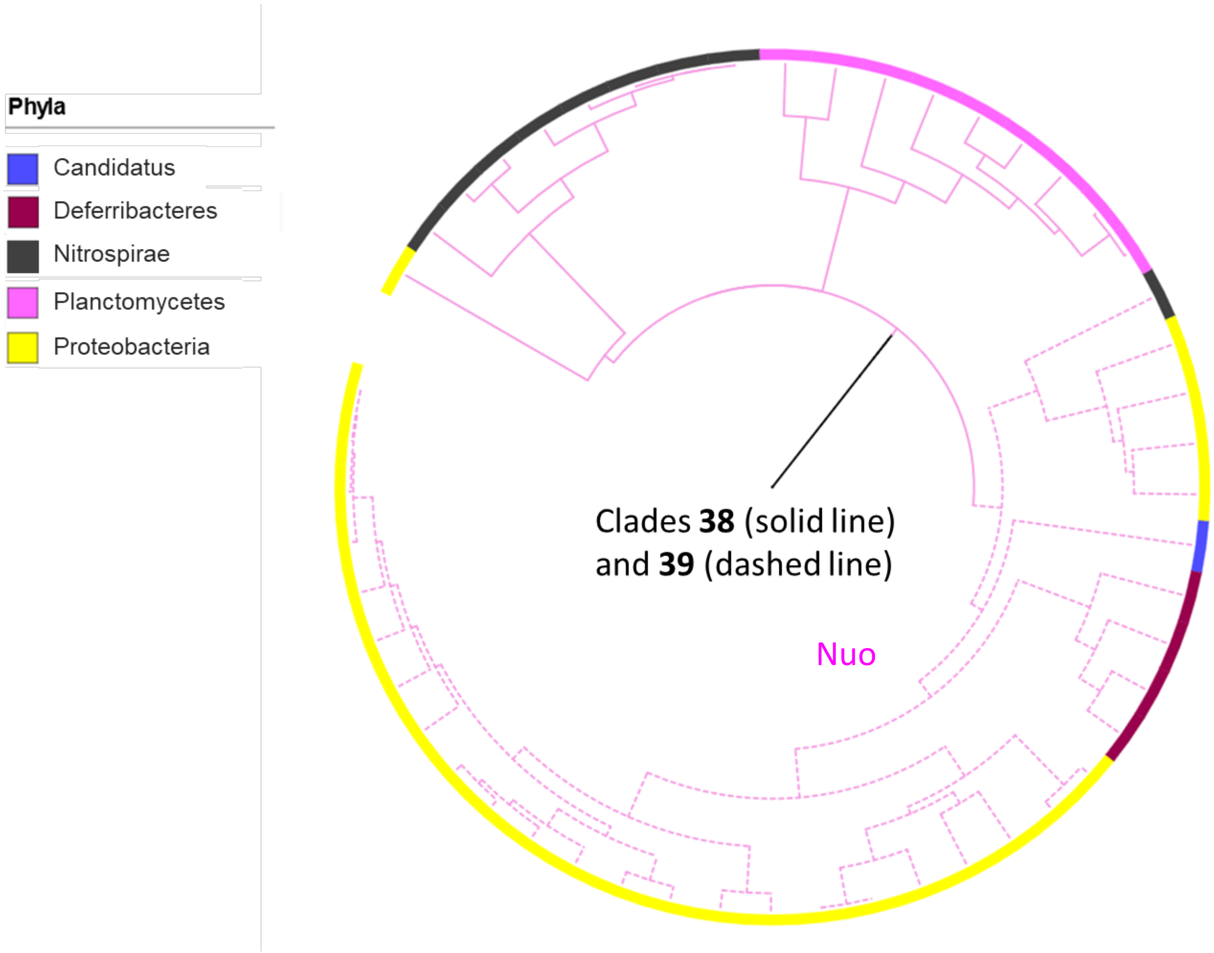

Supplementary Figure 6. Phyla associated with clades 38 (solid line) and 39 (dashed line) of the BfuB phylogenetic tree (see Figure 2). Both clades contain the Nuo-enzyme, separated into Deferribacteres and Proteobacteria (clade 38, 26; Nuo) and Planctomycetes and Nitrospirae (clade 39, 16; Nuo). In addition, clade 38 contains 4 Fdh and clade 39 contains 1 Fdh.

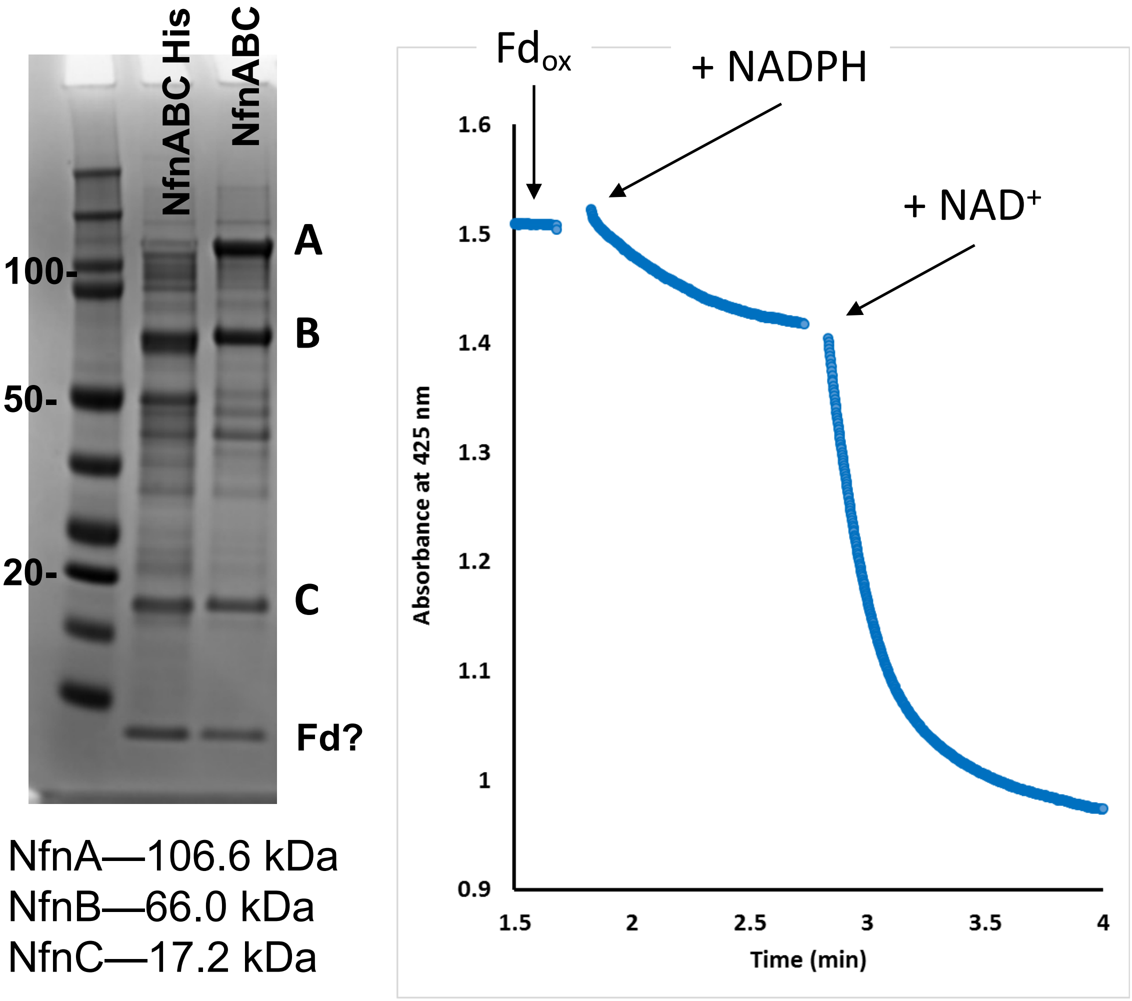

Supplemental Figure 7. *Left*: SDS-PAGE gel of *T. sibiricus* Nfn-Bfu purified from the hyperthermophile *Pyrococcus furiosus* after heterologous expression. NfnA (106.6 kDa), NfnB (66.0 kDa), and NfnC (17.2 kDa) subunits are indicated. Ladder sizes (in kDa) are indicated for select bands. *Right*: Assay progress curve showing bifurcating activity (20 U/mg) of *T. sibiricus* Nfn-Bfu. Fd_{ox}, NADPH, and NAD^+ were added where indicated. The activity only proceeds in the presence of all three substrates.

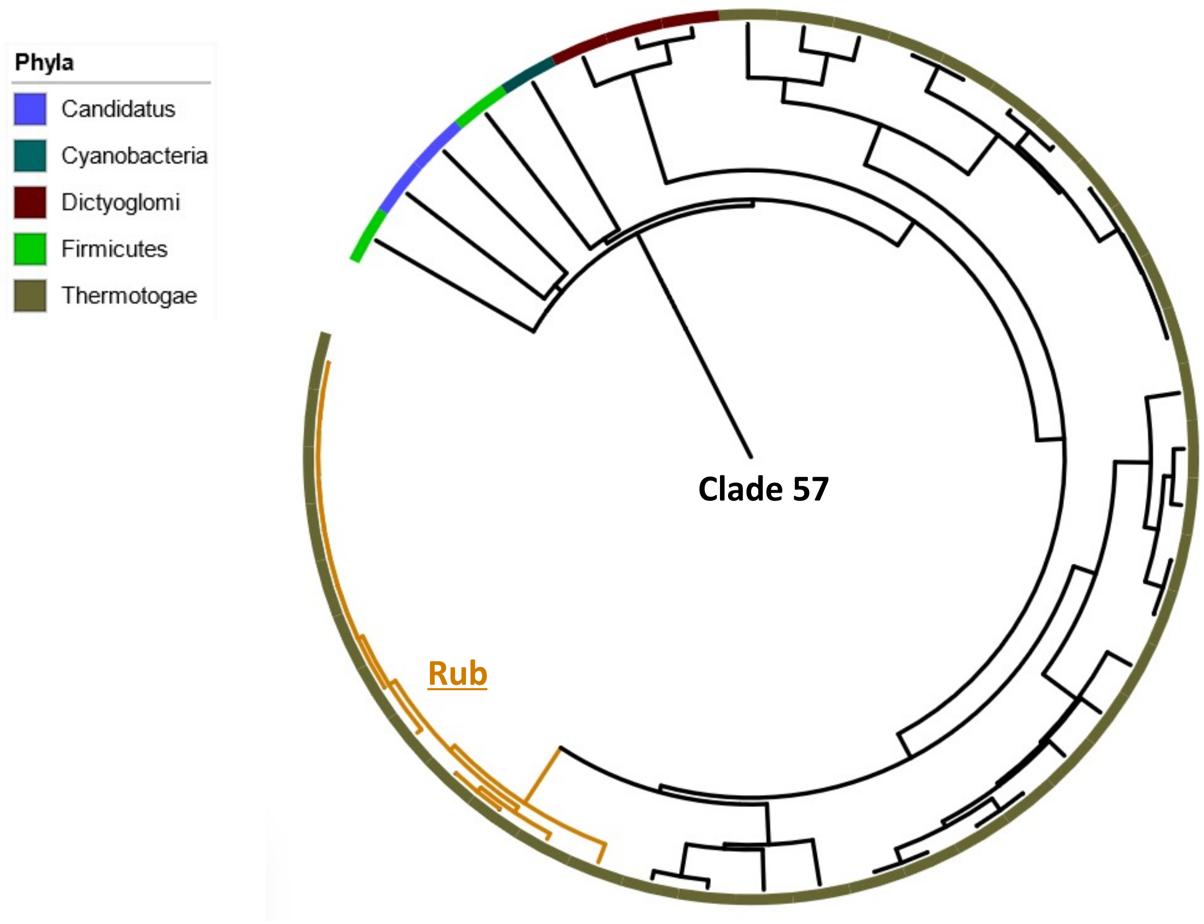

Supplementary Figure 8. Phyla associated with clade 57 of the BfuB phylogenetic tree (see Figure 2). The positions of the Rub (brown) enzymes are indicated

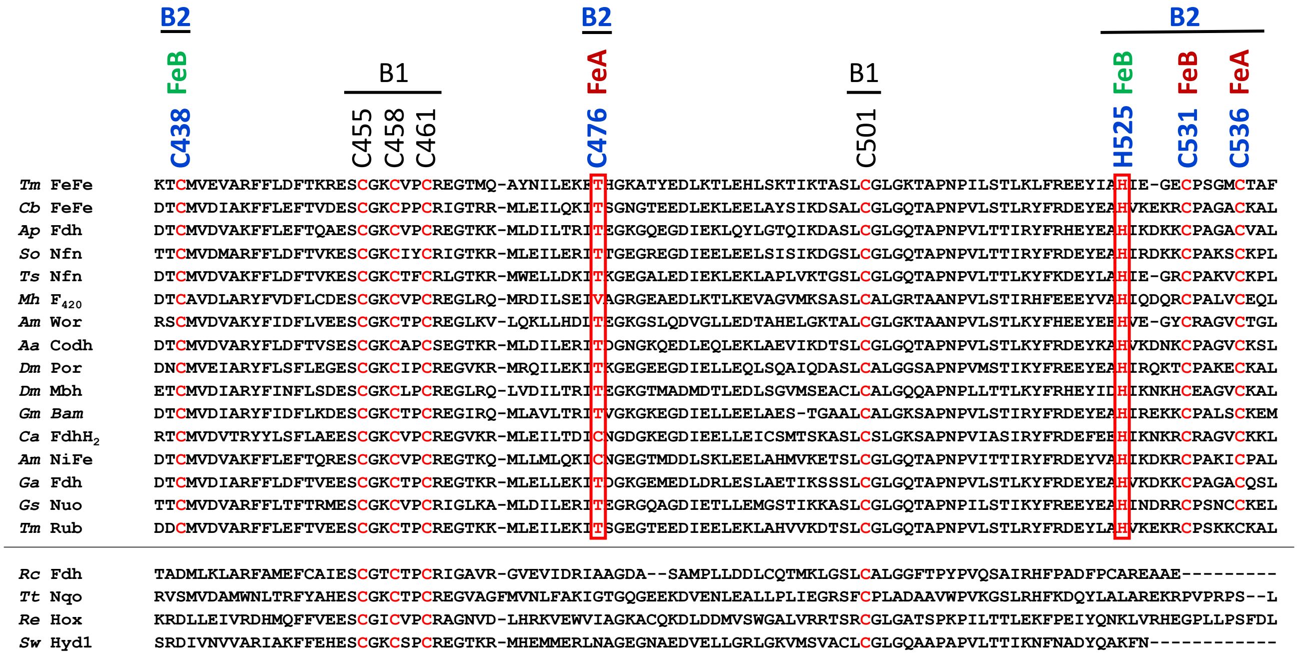

Supplementary Figure 9. Sequence alignment showing coordination of B1 and B2 clusters. Residues highlighted in red coordinate the indicated cluster. The horizontal line separates Bfu family enzymes (top) from homologous non-bifurcating complexes (bottom). The amino acid numbering is based on *A. mobile* NiFe-BfuB. While the three Cys (438, 531 and 536) and one His (525) coordinating the B2 cluster are conserved in all 1,558 Bfu sequences examined, the distribution of residues equivalent to Cys276 was as follows: 1055 Thr, 205 Cys, 195 Val, 55 Ser, 22 Ile, 10 Ala, 5 Leu, 4 Lys, 3 Met, 3 Pro, 1 Asp, 1 Glu and 1 Arg. The two Fe atoms of the B2 cluster are designated FeA (coordinated by two Cys) and FeB (coordinated by two Cys and a His). Abbreviations for organisms and enzymes are given in the text.

	A202 M204	F282
Tm FeFe	VCNGDEGDPGAFMNRT	KEGAGAFVCGEETALL
Cb FeFe	VCNADEGDPGAYMDRS	RLGAGAFVCGEETALM
Ap Fdh	ICNADEGDPGAFMDRS	KEGAGAFVCGEETALM
So Nfn	VCNADEGDPGAFMDRS	KKGAGAFVCGEETALI
Ts Nfn	VCNADEGDPGAFMDRN	KEGAGAFVCGEETALI
Mh F420	IVNCDEGDPGAFMDRA	HRGAGAFVSGESTALM
Am Wor	IANGDEGDPGAFMDRS	CKGGGAFVCGESSALM
Aa Codh	ICNADEGDPGAFMDRS	KEGAGAFVCGEETALI
Dm Por	ICNCDEGDPGAFMDRS	NRGGGAFVCGESTALM
Dm Mbh	LVNCDEGDPGAFMDRS	HEGAGAFVSGESSALM
Gm Bam	IVNADEGDPGAFMDRA	HMGAGAFVCGESSALM
Ca FdhH2	VCNGDEGDPGAFMDRS	VRGGGAFVCGESTALM
Am NiFe	ICNADEGDPGAFMDRS	KEGAGAFVCGEETALM
Ga Fdh	LCNADEGDPGAFMDRS	RLGAGAFVCGEETALI
Gs Nuo	ICNADEGDPGAFMDRS	KKGAGAFVCGEETALM
Tm Rub	VCNADEGDPGAFMDRS	RIGAGAFVCGEETALM
Rc Fdh	VCNVDEGDSGSFADRM	RVGAGAYVCGEETSLL
Tt Nqo	ICNADESEPGSFKDRY	HRGAGAYICGEETALM
Re Hox	ICNADEGEPGTFKDRV	QMGAGAYICGDESALI
Sw Hyd1	VCNADEGEPGTYKDRI	RMGGGAYVCGEESALI
	NADH Binding Domain	FMN Binding Domain

Supplementary Figure 10. Conservation of the residues within the FMN and NADH binding domains in BfuB. The amino acid numbering is based on *A. mobile* NiFe-BfuB. Non-bifurcating enzymes are shown below the horizontal line. Abbreviations are given in the text.

Activity	Clade	Type	Subunit Composition	Third Reaction	*E*º (mV)	Dominant Phyla	# of Members
Codh	49	2	Codh-Bfu**ABC**DEE	$CO + H_2O \rightleftharpoons CO_2 + 2\ H^+\ 2e$-	-520	Firmicutes	4
FdhFeFe	45	2/3	FdhFeFe-Bfu**ABC**DE/FS	$H_2 \rightleftharpoons 2H^+ + 2e^-$; $HCOO^- \rightleftharpoons CO_2 + 2e^-$	-421; -421	Firmicutes	10
F_{420}	45	1	F_{420}-Bfu**ABC**	$F_{420}H_2 \rightleftharpoons F_{420} + 2e^-$	-340	Euryarchaeota	3
Fdh	22, 23, 49, 53, 54, 56	1,2,3	Fdh-Bfu**ABC**	$HCOO^- \rightleftharpoons CO_2 + 2e^- + H^+$	-421	Euryarchaeota, Firmicutes, Proteobacteria, Chloroflexi, Synergistetes	82
FeFe	25, 54, 55, 56	1	FeFe-Bfu**ABC**(D)	$H_2 \rightleftharpoons 2H^+ + 2e^-$	-421	Firmicutes, Bacteroidetes, Thermotogae	957
Mbh	45	2	Mbh-Bfu**ABC**DE FGHI	$H_2 \rightleftharpoons 2H^+ + 2e^-$	-421	Chloroflexi	10
Nfn	53	1	Nfn-Bfu**ABC**	$NADPH \rightleftharpoons NADP^+ + 2e^-$	-324	Euryarchaeota, Firmicutes	140
NiFe	19,49	3	NiFe-Bfu**ABC**SL	$H_2 \rightleftharpoons 2H^+ + 2e^-$	-421	Synergistetes, Euryarchaeota	6
Nuo	38,39	3	Nuo-Bfu**ABC**DE FGHIJKLMN	$MQH_2 \rightleftharpoons MQ + 2e^-$	-74	Deferribacteres, Proteobacteria Planctomycetes, Nitrospirae	51
Por	35,44,45	2	Por-Bfu**ABC**DEFG	Pyruvate + CoA $\rightleftharpoons$ CO_2 + Acetyl-CoA+ $2e^-$	-500	Chloroflexi, Proteobacteria	12
Rub	57	4	Rub-BfuR**BC**	$H_2O_2 + 2H^+ + 2e^- \rightleftharpoons 2\ H_2O$	+1349	Thermotoga	13
Wor	45	2	Wor-Bfu**ABC**DE	$RCHO + H_2O \rightleftharpoons RCOO^- + 3H^+ + 2e^-$	-580	Synergistetes, Firmicutes	37

Supplementary Table 1. The types of enzyme within the Bfu family and their characteristics. Characterized and predicted enzymes and their microbial sources are shown in black and red font, respectively.
